# Media ownership and ideological slant: Evidence from Australian newspaper mergers

**DOI:** 10.1371/journal.pone.0315137

**Published:** 2024-12-31

**Authors:** Maxim Ananyev, Ekaterina Volkova

**Affiliations:** 1 Melbourne Institute, Melbourne University, Parkville, VIC, Australia; 2 Department of Finance, Melbourne University, Parkville, VIC, Australia; Universite Paris Pantheon-Assas, FRANCE

## Abstract

This study examines how media acquisitions by corporate conglomerates influence the political slant of Australian newspapers. Using a comprehensive measure based on the language of over 30 million news articles from more than 200 newspapers, we analyze the impact of three major acquisitions between 2016 and 2019. Employing a synthetic difference-in-differences approach, we find that acquisitions can significantly shift newspapers’ political slant, with effects varying by acquiring entity. News Corp’s 2016 acquisition led to a marked conservative shift in newspapers it already owned, while subsequent acquisitions by Nine Entertainment and the acquisition of Australian Community Media by Antony Catalano and Alex Waislitz resulted in shifts away from conservative slant in acquired newspapers. Our results provide evidence for the importance of supply-side factors in determining media slant, particularly in an era of increasing media ownership concentration.

## Introduction

Media concentration and pluralistic coverage of political events are cornerstones of a functioning society [1]. However, there is a global trend of mergers and acquisitions in the media market [2, 3]. According to a Gallup poll, more than 90% of the US citizens are concerned that local newspapers might reflect the views of new owners after acquisitions [4]. In our paper, we explore this concern, focusing specifically on the Australian media market for printed newspapers, which has recently experienced three major acquisitions by different conglomerates. Our analysis is based on 30 million news articles from more than 200 different newspapers. Our main contributions are twofold: first, we estimate and validate the measure of media political slant—the bias in news coverage favoring certain political ideologies or narratives over impartial reporting. Second, we evaluate how this measure of media slant changes after acquisitions.

Media slant is consequential for the economy, influencing political behaviour [5–7], firm valuations [8], and multiple personal decisions [9]. Even in the competitive systems of the developed world, many popular media outlets offer slanted news coverage [10–12]. In this paper, we explore the question of what drives media slant.

From a theoretical point of view, scholars explain the slant with two competing views: demand-side and supply-side [13]. In the demand-side explanation, the slant can be driven by the demand from the readers who would like to receive the coverage which aligns with their political dispositions. Thus, a profit-maximizing firm would offer a political slant in a bid to win more readers. In the supply-side explanation, the firms deviate from profit-seeking. Instead, they might have other goals, such as influencing the opinion of the readers. Because of that, owners, editors, and journalists shift their coverage towards a specific side of the political spectrum.

In this paper, we focus on the Australian newspaper market, which is notable for having the second highest concentration of media ownership in the world [14]. The sector underwent significant restructuring due to three major mergers in the late 2010s, which reshaped the industry. The potential link between media ownership and biased political news coverage is a significant concern in Australia. This issue has drawn considerable public attention, exemplified by a petition launched by former Prime Minister Kevin Rudd, which garnered over 500,000 signatures, calling for an investigation into the impact of media ownership [15]. The rise in media ownership concentration is a global phenomenon [16, 17], with similar efforts to examine its effects taking place in other countries as well [18]. Therefore, the Australian case can provide valuable insights and predictions regarding media trends in other developed countries.

For a sample of more than 200 Australian newspapers and 30 million news articles, we construct a time-varying measure of media bias by comparing the language of the news articles with the speeches of members of Parliament, following the methodology of [19]. We validate our measure of slant with a survey. Using this measure and a synthetic difference-in-differences approach [20], we estimate the effect of the change in ownership on the newspapers that were acquired. When possible, we also explore how the changes in trajectory of newspapers that were already part of the conglomerate before the merger to estimate how changes in market power affect existing newspapers.

Our preferred method relies on creating a synthetic counterfactual for the affected newspapers and then estimating the treatment effect using the difference-in-differences specification. We also control for the voting record in the regions of circulation to consider the demand-side explanations of bias.

Australia offers a unique opportunity to study the question of the effect of change in ownership on the media slant due to several factors. First, Australia is a developed economy with almost universal literacy, with 49% of citizens regularly learning news from newspapers [21]. Second, since 2010, Australia saw a notable merger & acquisition activity in the media industry. In particular three major deals significantly reshaped the print media landscape: (i) at the end of 2016, News Corp acquired Australian Regional Media including 49 of their regional newspapers, (ii) late 2018, two large media companies, Nine Entertainment and Fairfax Media merged, which resulted in Nine Entertainment acquiring control over some of the oldest and most popular Australian newspaper (including *The Age* and *Sydney Morning Herald*) and an array of regional newspapers, (iii) in 2019, Australian entrepreneurs Anthony Catalano and Alex Waislitz bought Australian Community Media from Nine Entertainment. We are able to track the context of the affected and unaffected newspapers before and after the deals.

Our results provide a nuanced view of the impact of ownership changes on media slant. First, we examine the effect of News Corp’s acquisitions of regional newspapers in New South Wales and Queensland. We find no discernible impact on the slant of the acquired newspapers. However, newspapers already controlled by News Corp before the acquisition show a significant shift towards a conservative slant. This shift is strongly statistically significant across nearly all regions, with a magnitude equivalent to 26% of the standard deviation. Second, we observe that subsequent mergers shifted the political slant of acquired newspapers away from the conservative side. Specifically, the Nine Entertainment acquisition resulted in a shift corresponding to 34% of the standard deviation, while the ACM acquisition led to a 16% shift. In summary, our findings indicate that mergers tend to push the political slant of newspapers in varying directions, depending on the acquiring entity.

Our results might be rationalised with the following considerations: media consumers actually prefer the diverse media landscape, but as the ownership becomes more concentrated, supply-side factors prevail. This is why News Corp media could have shifted to the right when the competition decreased following the mergers. Two of the consequent media mergers decreased the concentration of ownership of the regional newspapers, introducing more competition to what has previously been almost a duopoly of two major media conglomerates, Fairfax and News Corp. This acquisition increased the competition thus also increasing the importance of demand-side factors.

Our study contributes to the literature on the origins of media bias. The closest paper to ours is [19] where the authors study potential media slant of daily newspapers in the US. They find the effect of supply-side factors to be negligible. Our results are different and yet nevertheless consistent with [19]. Because [19] study newspaper up to year 2005, their sample does not include much of the subsequent period characterised by the newspaper decline largely driven by both readers and advertisers shifting to the new online platforms and social media. During this decline, more than 2,200 local newspapers were closed in the US, the number of professional journalists declined by more than a half between 2005 and 2021, many large newspaper chains filed for bankruptcy, newspaper revenue dropped by more than 50% between 2002 and 2021 [22]. Our results show that in this new economic reality, the supply-side factors play a larger role. More broadly, our paper is related to the theories of media slant [23–28]. We offer a new empirical test that allows to adjudicate between various hypotheses regarding the sources of the slant. We also emphasize that the theories of the slant should consider political and economic environment in which media outlets operate. In the context of Australia, [29] offered an estimation of media slant of several Australian newspapers based on their mentions of public intellectuals. Their conclusion was that Australia’s media was largely centrist. Our study explores the dynamic of media slant focusing on the impact of post-2012 acquisitions.

Another paper close to ours is [30] which also measures slant by comparing the newspaper content with the speeches of politicians. Focusing on the Swedish newspapers, it finds that the slants of newspapers owned by the same firm correlate strongly even if they circulate on markets with different ideological preferences of the audience. Our paper, focusing on Australia’s local newspapers, explores how the slant changes dynamically in response to the M&A activity of the media owners.

The study of the origins of media slant is of considerable academic interest. With the news consumption becoming more segregated [31] and well-documented persuasive effects of media [12, 32] understanding of the demand and supply of bias is crucial for understanding political and economic behaviour. Apart from the previously discussed [19, 30], the documented factors of media slant were political connections of management and owners [33], international trade negotiations [34], influence of network cable news on the local news [35], and ideology of the newsroom [36].

Our study has important policy implications. Robust independent media are important for political participation, good governance [37, 38] and seamless operation of markets [39], while the captured media can have detrimental effects [40]. It is thus in the public interest to ensure that the diversity of media. Our results are consistent with the view that increase in media ownership concentration is detrimental to the diversity of viewpoints.

## Data and methods

### Data

We use two primary data sources: parliamentary speeches by Australian politicians and newspaper articles from Australian print media. We download the transcripts of speeches from the Australian Parliament’s website, for the period from 2001 to 2022 [41]. The text of newspaper articles is obtained from ProQuest, which archives over 200 Australian newspapers between 2000 and 2022, resulting in more than 30 million news articles. Additionally, we manually collect information regarding the ownership of these newspapers.

Following [19], our analytical approach is divided into two parts. First, we identify politically slanted phrases within the Australian Parliamentary Speeches. Second, we use these phrases to estimate the political slant of Australian newspapers.

### Construction of slant measure

The first step of our analysis is to download and filter speeches, focusing exclusively on those associated with Australia’s two primary political parties: the Labor Party and the Coalition. The Labor Party typically takes a more pro-worker position and a more progressive social stance, while the Coalition, comprising the Liberal Party and the National Party, takes a more pro-business stance and is more aligned with conservative social values. We split the sample into three periods based on the ruling political party. The first period begins at the start of the sample in 2000 and ends in December 2007, a time interval during which the Coalition held a parliamentary majority under Prime Minister John Howard. During the second period, from December 2007 to September 2013, the Australian Labor Party was in control, with the prime ministerial role transitioning between Kevin Rudd, Julia Gillard, and returning to Kevin Rudd. The third period starts in September 2013 and continues until the end of the sample, throughout which the Coalition regained the majority, with Tony Abbott, Malcolm Turnbull, and Scott Morrison serving as Prime Ministers. In May 2022, the Coalition lost majority, and the new Labor government commenced with Anthony Albanese as Prime Minister. Each political period is characterized by distinct key topics of debate.

We analyze the texts of all public speeches by the two major parties in the parliament. We remove all the stopwords (articles, prepositions), apply a stemming procedure to all the words (so that words like “tax” and “taxes” are treated as the same word), and divide all the speeches into 3-word combinations (trigrams) and enumerate all these combinations.

For every 3-word combination in each out of three political periods, we calculate the *χ*^2^ measure, using the following formula:

$$\chi_{p}^{2}=\frac{{\left(f_{pc}f_{\tilde{pl}}-f_{pl}f_{\tilde{pc}}\right)}^{2}}{\left(f_{pc}+f_{pl}\right)\left(f_{pc}+f_{\tilde{pc}}\right)\left(f_{pl}+f_{\tilde{pl}}\right)\left(f_{\tilde{pc}}+f_{\tilde{pl}}\right)}$$

Here, *p* identifies the phrase, *f*_*pc*_ is the number of times the phrase *p* occurs in the speeches by Coalition MPs, $f_{\tilde{pc}}$ is the number of phrases uttered by Coalition MPs which do not include phrase *p*. Analogously, *f*_*pl*_ is the number of times the phrase *p* occurs in the speeches by Labor MPs, and $f_{\tilde{pl}}$ is the number of phrases uttered by Labor MPs which do not include phrase *p*.

Described *χ*^2^ scores are estimated for each trigram, with higher *χ*^2^ values suggesting a phrase’s prevalence within one of the political parties. We select phrases with the highest *χ*^2^ for each political period and assign them to a party based on relative frequency. Hence, if a phrase *p* has a high *χ*^2^ value and is more frequently used in speeches by Labor Party members than by Coalition members, we categorize it as a Labor phrase. Alternatively, if a phrase with a high *χ*^2^ value is mentioned more frequently by Coalition party members then it is a Coalition phrase.

In our analysis, we focus on the most common phrases associated with the Labor and Coalition parties, excluding trigrams that are names of legislative acts, Parliamentary procedures, government agencies, and general phrases. We then narrow down each of our six lists (2 parties × 3 periods) to include only the top 150 3-word combinations with the highest *χ*^2^ scores. The full list of all trigrams is available on request and can be used to estimate the political slant of other Australian news sources. The cut-off of 150 trigrams per period per party was selected to balance the need for the trigrams to be sufficiently distinctive while also covering the wide variety of topics. Supplementary Material provides robustness checks of our main estimations with 50, 100, and 200 trigrams per party per period.

Table 1 presents examples of the top-15 Labor/Coalition trigrams for each period. During the first period (January 2000—December 2007), the most prevalent phrases for the Labor party were pharmaceutical “benefits scheme”, “make ends meet”, “paid maternity leave”, “credit card debt”, and “ratify Kyoto Protocol.” Conversely, the Coalition party frequently mentioned “unfair dismissal laws”, “embryonic stem cell research” and “strong economic growth”. These phrases indicate that each party focuses on unique topics, such as embryonic cell research for the Coalition and maternity leave for Labor. More importantly, however, is that the parties often discuss the same issues using different terms. For example, the Labor party frames economic discussions with phrases like “make ends meet,” whereas the Coalition uses terms like “strong economic growth”. This trend continues across other periods in our sample. For instance, in the second period, the Labor party discusses education, while the Coalition focuses on security (“outlaw motorcycle gangs”) and immigration (“temporary protection visas”). Nevertheless, both parties engage in environmental discussions, albeit with varying emphases. The Labor party places a stronger emphasis on climate change, using phrases such as “action against climate change”, “tackle climate change”, “rising sea levels,” and “effects of climate change.” Meanwhile, the Coalition highlights the economic implications of climate policies, with phrases like “cost of carbon tax”, “increase in electricity prices”, and “new carbon tax”. The third period continues the trends described earlier: each party maintains focus on its unique topics, such as racial discrimination and domestic violence for Labor, and infrastructure projects (e.g., “black spot program”) and the decrease of bureaucracy (e.g., “cutting red tape”) for the Coalition. However, the parties substantially differ in their discussion of economic issues. The Labor party places a stronger emphasis on consumers, using phrases like “make ends meet” and “struggle to make ends meet”, while the Coalition shifts its focus toward small businesses, with trigrams such as “Australian small business” and “family business enterprise”.

**Table 1:** Most common labor/coalition trigrams. This table presents examples of trigrams from parliamentary speeches that are highly characteristic of the Labor and Coalition parties. Subtable (a) displays the fifteen trigrams with the highest Labor-aligned chi-squared values for the periods 2000–2007, 2008–2013, and 2013–2022. Correspondingly, subtable (b) lists the fifteen trigrams with the highest chi-squared values for the Coalition across the same time spans. All words within the trigrams have been stemmed.

(**a**) Labor Trigrams			
**No.**	**2000–2007 Period**	**2008–2013 Period**	**2013–2022 Period**
1	Pharm. Benefits Scheme	coal seam gas	make ends meet
2	make ends meet	Building Education Revolution	action climate change
3	paid maternity leave	action climate change	impact climate change
4	credit card debt	occupational health and safety	struggle make end
5	ratify Kyoto protocol	tackle climate change	tackle climate change
6	struggle make end	renew energy target	put food table
7	pressure interest rate	rising sea level	racial discrimination act
8	tackle climate change	sea level rise	clean energy finance
9	full sale Telstra	impact climate change	invest renew energy
10	rise sea level	seam gas mine	family domestic violence
11	effective marginal tax	effect climate change	live poverty line
12	medicare safety net	extreme weather event	Uluru statement heart
13	private health fund	clean energy future	support marriage equality
14	health care system	trade training centre	climate change energy
15	nuclear waste dump	carbon capture storage	act climate change
(**b**) Coalition Trigrams			
**No.**	**2000–2007 Period**	**2008–2013 Period**	**2013–2022 Period**
1	unfair dismissal law	outlaw motorcycle gangs	small medium business
2	embryonic stem cell	big new tax	mental health suicide
3	loan interest rate	man woman child	Australian small business
4	strong economic growth	every man woman	Black Spot program
5	adult stem cell	private health cover	small business family
6	home loan interest	economic fiscal outlook	mobile Black Spot
7	new job creation	electricity price rise	cut red tape
8	protect nuclear safety	cost carbon tax	health suicide prevention
9	radiation protect nuclear	health insurance premium	business family enterprise
10	Australian radiation protect	business consumer confidence	small medium business
11	unfair dismissal claim	carbon tax cost	Australian energy regulation
12	natural heritage trust	carbon tax mine	small family business
13	good economic management	increase electricity price	create local job
14	Australian protection svcs	new carbon tax	new job create
15	state government response	carbon tax go	instant asset write-off

The second step of our analysis involves estimating the political slant of newspapers over a given period through the use of 150 phrases associated with the Coalition/Labor. This is conducted on a sample of Australian newspapers sourced from ProQuest. Our dataset encompasses between 100 and 300 newspapers depending on the period. The sample size increased significantly as various digital versions of newspapers were incorporated into the ProQuest library around 2010. A subsequent decline in the number of newspapers after 2020 can be attributed to the closure of several regional newspapers [42]. The total volume of news articles ranges between 3,500 and 10,000 per quarter.

We use texts of all news articles to calculate the frequency of each political trigram from the previous step and estimate the political slant of newspaper *n* for month *t* according to the following formula:

$$\begin{array}{c} Slant_{n,t}=\frac{nlp_{n,t}-ncp_{n,t}}{nnews_{n,t}} \end{array}$$

In the equation above, *nlp*_*n*,*t*_ represents the number of top-150 Labor trigrams mentioned in all news articles of newspaper *n* in month *t*. Similarly, *ncp*_*n*,*t*_ stands for the number of top-150 Coalition trigrams in all articles of newspaper *n* in month *t*. Lastly, *nnews*_*n*,*t*_ corresponds to the total number of news articles in newspaper *n* during month *t*. Higher values of *Slant*_*n*,*t*_ correspond to a greater alignment of newspaper articles with Labor politicians.

**Fig 1:**
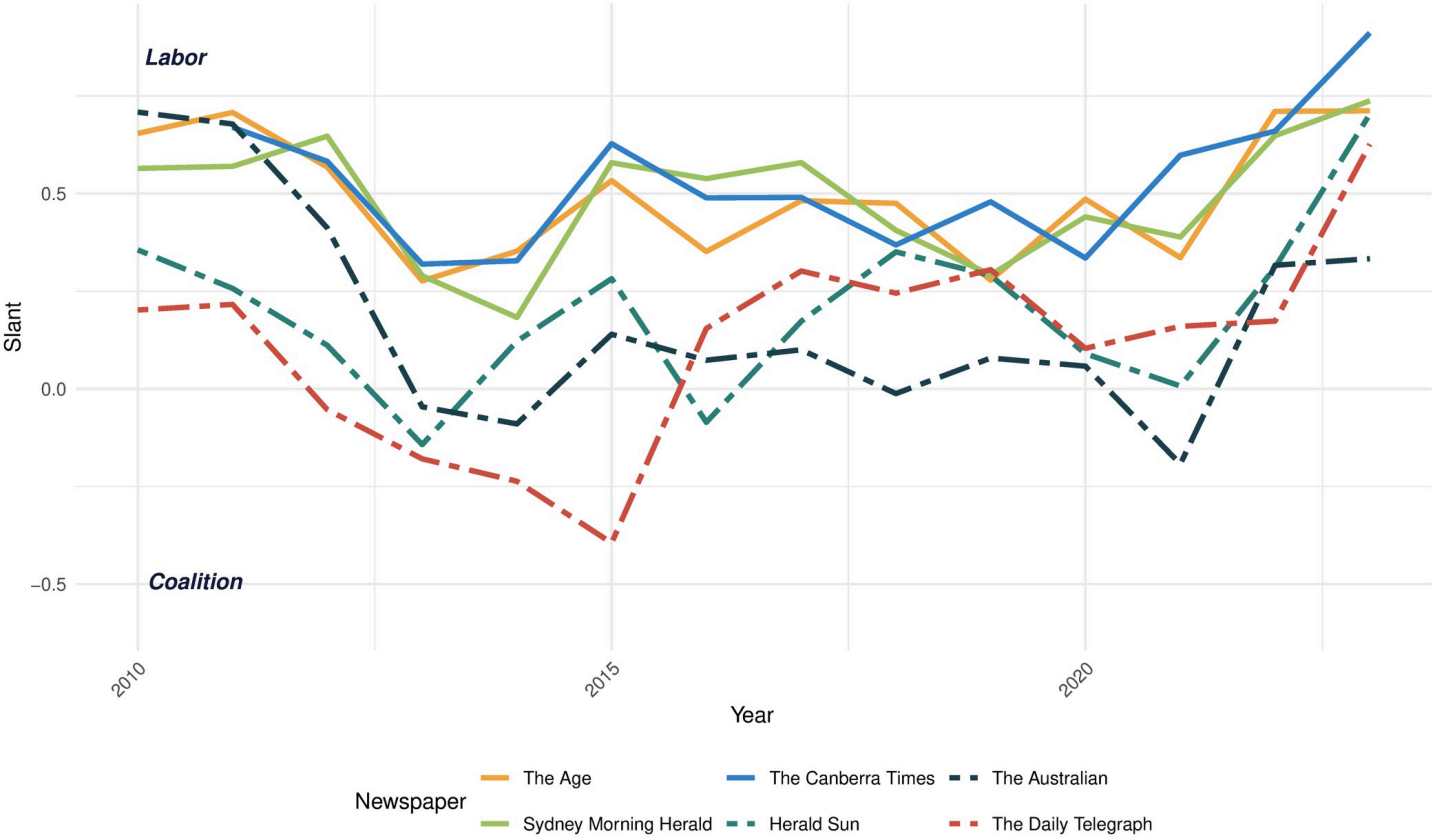
Political slant of selected newspapers. This figure depicts the trend in political slant across six popular newspapers, with a detailed description of the political slant. Solid lines represent newspapers that belonged to Fairfax Media in 2010, whereas dashed lines denote newspapers that were part of the News Corp conglomerate.

Fig 1 shows the trend in political slant among six major newspapers in our study: *The Age*, *Sydney Morning Herald*, *The Canberra Times*, *Herald Sun*, *The Australian*, and *The Daily Telegraph*. These newspapers cater to audiences in different parts of the country and also vary in their ownership. For example, *The Age* and *Sydney Morning Herald* were formerly owned by Fairfax Media but were acquired by Nine Entertainment in 2018. Similarly, *The Canberra Times* was also a part of Fairfax Media but was purchased by the Australian Community Media Group in 2013. The last three newspapers: *Herald Sun*, *The Australian*, and *The Daily Telegraph* have been under News Corp’s ownership throughout the years covered in the graph. Despite differences in target audiences and ownership, political slants of the newspapers have similarity in trends. For instance, all newspapers showed a tendency to lean more towards the Coalition between 2010 and 2014, with a marked increase in pro-Labor slant in recent years across all six publications. However, the three newspapers that were originally part of Fairfax Media have consistently exhibited a more pro-Labor political slant throughout most of the years, in contrast to the three newspapers owned by News Corp.

### Verification of the measure

**Fig 2:**
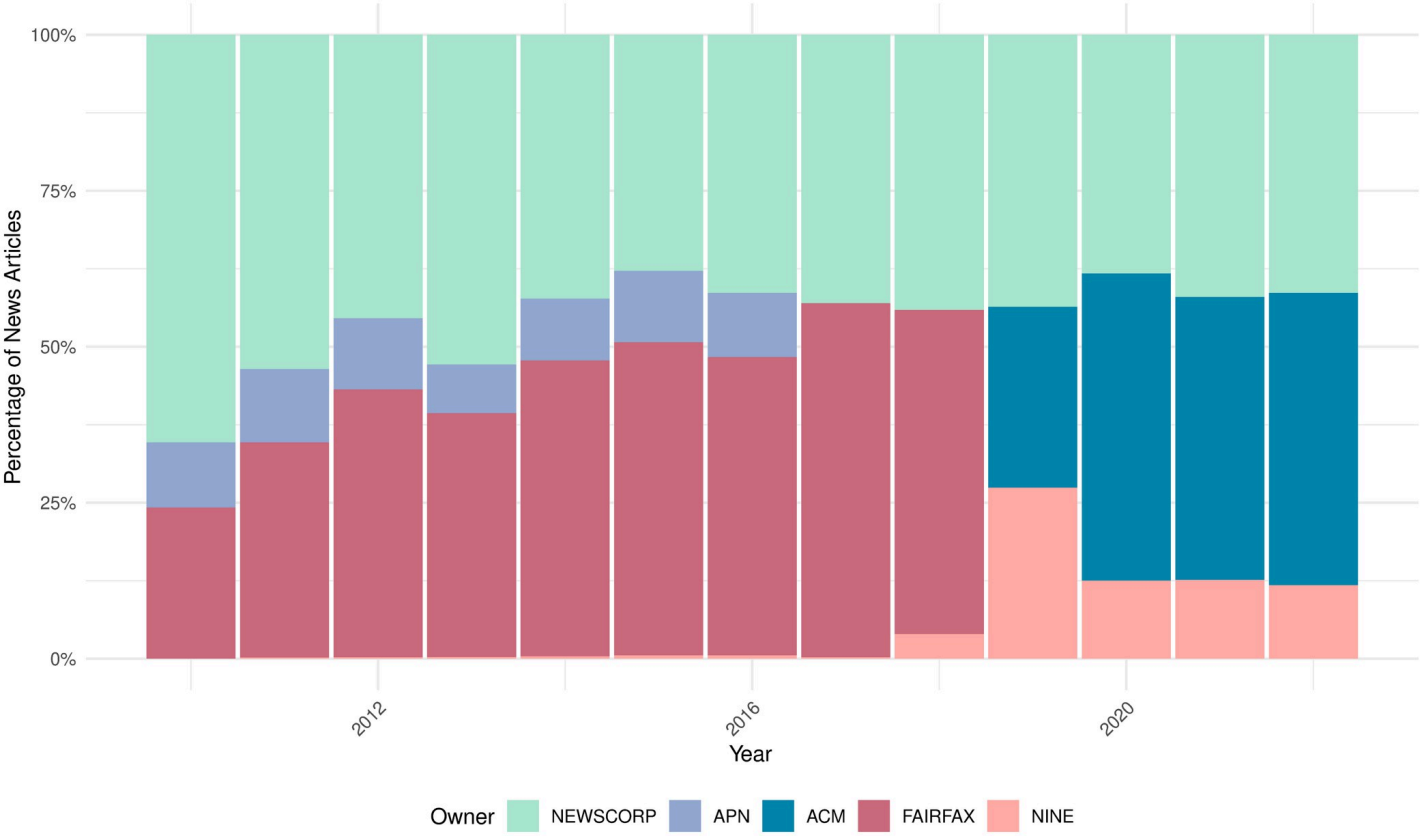
Political slant measure verification. This figure provides validation for proposed measure of political slant. The validation process is based on the data from a Melbourne Institute survey carried out in December 2023. The first panel depicts the relationship between our political slant measure on the Y-axis and the survey participants’ preferences for the Labor party on X-axis. Labor preferences among newspaper readers were quantified as the ratio of readers who are aligned or strongly aligned with the Labor party and who read the newspaper because it aligns with their views to the total readership. Each circle on the graph represents one of the newspapers; the size of the circle indicates the total readership within the survey sample, and the red dashed lines depict a fitted OLS regression line. In a similar vein, the Coalition bias is determined by the ratio of readers whose views are aligned or strongly aligned with the Coalition party to the overall number of readers. The total bias is calculated by deducting the Coalition bias from the Labor bias.

To validate our political slant measure we commissioned a survey with the Melbourne Institute and Roy Morgan Research [21]. This survey, based on a representative sample of 1,025 Australians, asks participants about their readership of the most popular newspapers and, for each newspaper they read, the survey asks if they read it *because* that newspaper aligns with their views. In a separate question, the respondents are asked about their voting intention. Thus, for every newspaper we construct its Coalition bias as a share of readers who are Coalition voters reading the newspaper *because* it aligns with their views. Analogously, we construct the Labor bias as a share of readers who are Labor voters reading the newspaper because it aligns with their views. Fig 2 presents a scatterplot where our political slant measure is plotted against the survey outcomes.

The first panel compares the average political slant (Y-axis) with the Labor bias among newspaper readers. The dashed red lines in the panel depict the fitted values from an OLS regression. This panel demonstrates a strong correlation between our measure of political slant and Labor bias calculated with the survey with a correlation coefficient of 0.50. Similarly, the second panel examines the relationship between the average political slant of newspapers and the survey-based measure of a Coalition slant. The correlation coefficient for these measures is -0.66.

Lastly, the third panel synthesizes the findings from the first two panels, comparing the political slant of newspapers with the total bias of their readers, where total bias is defined as the Labor bias minus the Coalition bias. The correlation between the newspapers’ political slant and the total bias of their readers is 0.72. High value of correlation between our measure and the survey data provides a validation for our approach.

It’s also important to point out that the dynamics of the slant is consistent with the notable changes in Australia’s media landscape. First, we see the gradual movement of *The Australian* to more and more conservative positions between 2010 and 2021. This is consistent with the assessment of former Australian Prime Minister Kevin Rudd, who observed, “If you look at Murdoch’s election “news” coverage during the 2007 campaign, it was at best 50–50. Roll the clock to 2010 and 2013, it was more like 80–20 against Labor. Then 90–10 in 2016. And in 2019 it was 100–0” [43].

Another important feature of the observed dynamics is the left-ward shift of *The Australian* in 2021 as well as the other newspaper around 2019–2020. This is consistent with the increased salience of climate change in Australia following the 2019 election and the bushfires of late 2019—early 2020, as well as a well-documented editorial shift in the News Corp newspapers regarding the acceptance of the reality of man-made climate change [44].

A seemingly counterintuitive aspect of the estimated media slants is that *The Australian* in the beginning of the period looks Labor-slanted. It’s worth pointing out that we omit the op-eds and only focus on the news articles. This result is consistent with the estimates by [29] who showed that *The Australian* with the op-eds was Coalition-slanted, but *The Australian* without the op-eds was the most left of the centre newspaper among the ten major Australian newspapers they analysed. We see this as an additional validation of our result, since the method in [29], which focused on the mentions of public intellectuals, is significantly different from ours.

### Mergers

**Fig 3:**
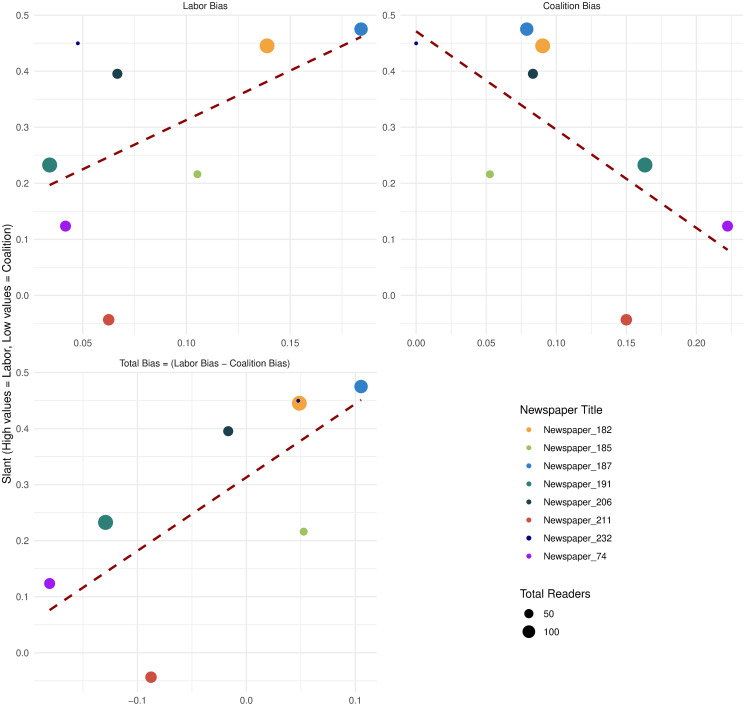
Australian media ownership. This figure shows the ownership composition of the newspapers in our sample, with different colors indicating the total percentage of newspapers owned by each conglomerate. Ownership is determined based on the ultimate parent company, defined as any entity owning more than 50% of a newspaper. **NEWSCORP** represents News Corp-owned newspapers. **APN** refers to newspapers previously under APN News & Media, acquired by NewsCorp in 2016. **FAIRFAX** denotes Fairfax Media, **NINE** represents Nine Entertainment Co., which acquired Fairfax Media. **ACM** refers to Australian Community Media.

Australia has one of the highest levels of media ownership concentration globally [14, 45]. Fig 3 shows the concentration of newspaper ownership. The majority of Australian newspapers belong to one of the 2–3 conglomerates over time. Throughout the whole sample period, News Corp owned around half of the sample newspapers. In the first part of the sample, Fairfax Media controlled 25% to 50% of newspapers. Media acquisitions by Nine Entertainment as well as Catalano and Waislitz changed the ownership composition. This study examines the political slant shifts in Australian newspapers following three major acquisitions:

1. **News Corp’s Acquisition of APN News & Media (2016):** Over 60 regional newspapers in NSW and QLD, including *The Gympie Times* and *The Northern Star* [46, 47].
2. **Nine Entertainment’s Acquisition of Fairfax Media (2018):** 117 newspapers, including *The Age* and *The Sydney Morning Herald* [48, 49].
3. **Catalano and Waislitz Acquisition of Australian Community Media (2019):** 102 newspapers, including *Advocate* and *The Canberra Times* [50].

The landscape of Australian printed media has two unique features that allow us to conduct a cleaner analysis.

1. Even before the three mentioned acquisitions, Australian newspaper ownership was concentrated, mostly among conglomerates, with no significant share of independent media. Hence, in our analysis, we can estimate the effect of a change in ownership (conglomerate to conglomerate) rather than a change in ownership type (independent newspaper to conglomerate) as in [51].
2. Acquisitions affect newspapers across the political spectrum differently, allowing for an analysis of political slant changes following acquisitions by conglomerates with distinct political leanings.

News Corp is the largest owner of print media in Australia. Its proprietor and executive chairman (2013–2023) Rupert Murdoch is described as “staunch conservative” and the outlets associated with him are described to exhibit a leaning towards a conservative side of the political spectrum [12, 52].

Fairfax Media was one of the oldest media companies in Australia. In 2012, after the Fairfax family sold its stake, Gina Rinehart, Australian mining magnate known for her support for conservative politicians and opposition to carbon tax, became its largest shareholder [53–55]. She sold her Fairfax stake in 2015 [56]. Fairfax Media itself was not named as providing funds to political parties since 2012.

Nine Entertainment is a publicly traded company with a diverse set of shareholders. According to Australian Electoral Commission, in 2012–2019, Nine Entertainment was named as providing funds to Australian Labor Party (more than 50,000 AUD over 7 years) [57]. It should be also noted Fairfax journalists had adopted a charter asserting their independence [58]. It was also reported that the charter remained unsigned by Nine executives [59].

Australian Community Media is a media company responsible for a set of regional newspapers. It has been a part of Fairfax Media since 2007, then a part of Nine Entertainment (since the Nine/Fairfax merger in 2018). In 2018, it was bought and Antony Catalano and Alex Waislitz [60].

The new owners of Australian Community Media, Antony Catalano and Alex Waislitz, refrain from publicly expressing their political views, emphasising instead the business aspects in their appearances and interviews. Notably, Alex Waislitz’s philanthropic foundation has partnered with the Clontarf Foundation, an organisation dedicated to enhancing opportunities for Indigenous children [61]. It is also important to acknowledge that phrases surrounding Indigenous issues are associated with the speeches of Labor Party politicians. Neither Catalano nor Waislitz are shown on the returns of the Australian Electoral Commission as donors around the time of the acquisition.

### Empirical design

In this study, we investigate how the political slant of newspapers evolves following a change in ownership. While acknowledging the non-random nature of acquisition decisions, we aim to construct an appropriate counterfactual for the potential trend in newspaper slant. A newspaper’s political leaning is highly sensitive to the prevailing political climate, and as depicted in Fig 1, time trends exhibit certain commonalities across publications.

To construct a hypothetical continuation of a newspaper’s political slant trajectory, we employ the synthetic difference-in-differences (SDID) methodology proposed by [20]. This approach utilizes a pool of newspapers that did not experience an ownership change as potential control units. This method combines the benefits of synthetic control approaches [62] and traditional difference-in-differences estimation.

Essentially, synthetic difference-in-differences [20] is a weighted version of the standard two-way fixed effects estimator. It gives more weight to units (like newspapers) that have a similar past to the treated units, and it also highlights time periods that are similar to the treated periods. By focusing on units and periods that are alike, the estimator becomes more robust.

We construct a synthetic control unit by identifying a weighted combination of these control units that best replicates the pre-treatment characteristics of the treated newspaper (i.e., the newspaper that underwent an ownership change). The synthetic control construction allows us to optimally mimic the pre-treatment behavior of the treated group. Subsequently, we implement the difference-in-differences estimation between the treated newspaper and its synthetic counterpart.

## Results

**Fig 4:**
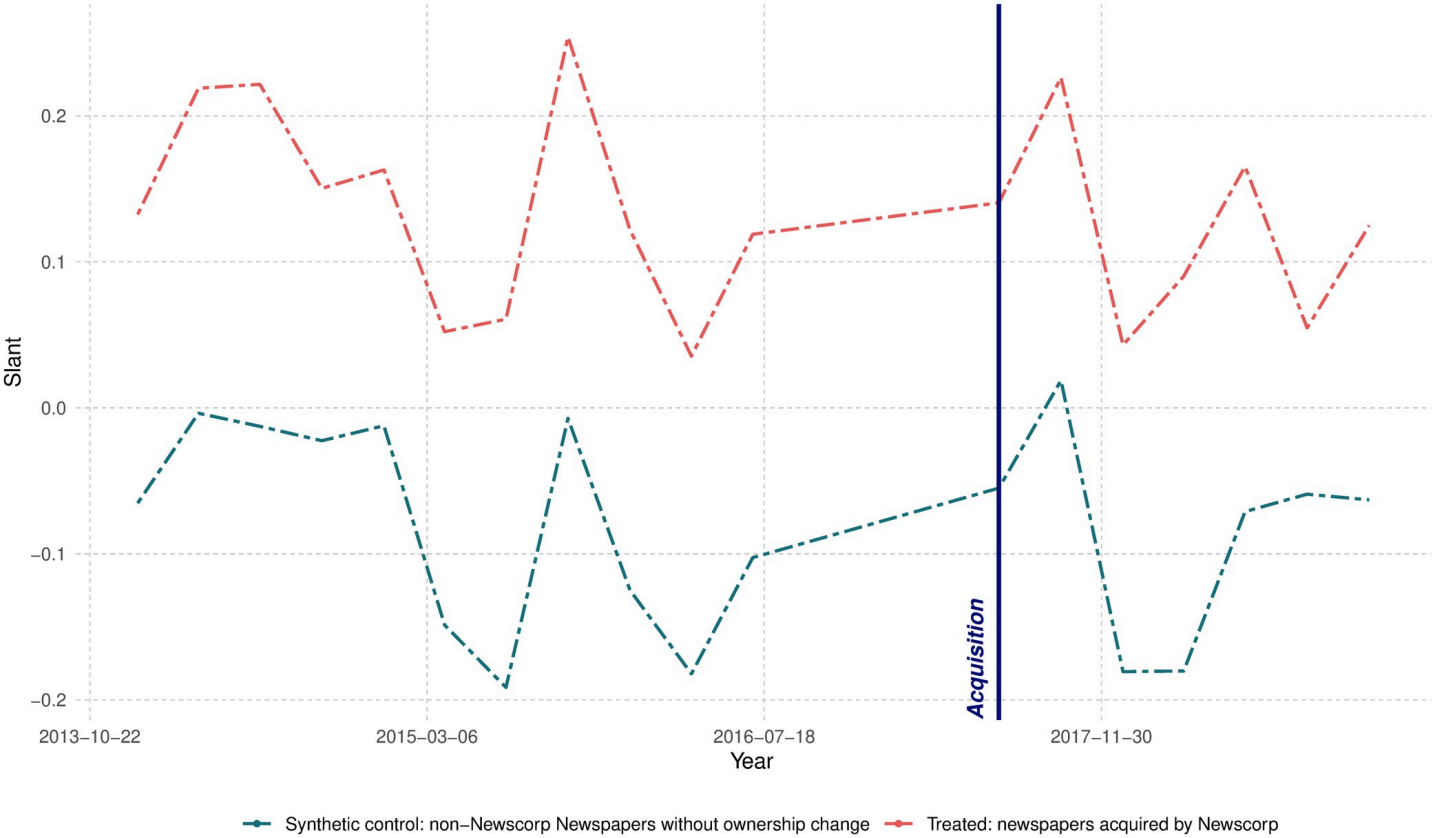
Change in political slant after News Corp acquisition. This figure illustrates the results of a Synthetic Difference-in-Differences (SDID) analysis. In this analysis, the control group consists of non–News Corp newspapers that remained under the same ownership, whereas the treatment group comprises newspapers acquired by News Corp prior to the 2016 acquisitions. The dot-dashed red line represents the average trend of the treated group, while the dot-dashed green line shows the average time trend for the synthetic control. Solid red and green lines depict the average linear trend between pre-treatment and post-treatment periods for the treatment and synthetic control groups, respectively. Small black error bars provide graphical estimates of the synthetic difference-in-differences effect. The vertical navy line indicates the first post-event observation.

The first merger in our analysis is News Corp’s acquisition of regional newspapers in New South Wales and Queensland. Eleven newspapers in our sample changed ownership after this acquisition, comprising our treatment group. The donor pool for our control group consists of newspapers that did not belong to News Corp and did not experience an ownership change. We employed a synthetic difference-in-differences approach for comparing the treatment and control groups. Specifically, we focused on the interval starting three years before the merger and ending two years after, excluding the quarter of the merger and the subsequent quarter from our analysis. We calculated the quarterly media slant for each newspaper in the treatment group and the control pool group. Then, we used the synthetic difference-in-differences method to construct a synthetic control group. We use synthdid package in R to conduct out analysis [63]. Fig 4 shows the results. According to the figure, the synthetic control group closely mimicked the political slant behavior of the treatment group. In the post-merger period, the similarity between trends continued, and a more detailed test for the difference between the treatment and control groups suggested no significant difference in the post-period. Specifically, the 95% confidence interval for the difference in media slant was [-0.133, 0.121]. Table 2 repeats the analysis on various subsamples: newspapers acquired in the states of NSW or QLD, or in the electoral regions with high support for Coalition (low Coalition support region small it too small for a reliable estimate). The table reports estimations based on the “jackknife” variance estimator in the synthetic difference-in-differences approach. Overall, all estimations suggest that we cannot conclude that News Corp’s acquisition changed the political leaning of the acquired newspapers.

**Table 2:** Shows the change in political slant after the News Corp acquisition. This table presents the estimates from a Synthetic Difference-in-Differences analysis, comparing trends between newspapers acquired by News Corp and a synthetic control group. The first row provides the point estimate of the difference, the second row shows the “jackknife” estimated standard errors, and the third row reports the total number of newspaper-quarter observations for both the treatment and control samples combined. Rows four to seven present summary statistics for the treatment sample during the pre-event window. Specifically, the fourth (sixth) row reports the 25th (75th) percentile, the fifth row shows the mean, and the seventh row provides the standard deviation. The first column displays results for the full sample, while the second and third columns focus on newspapers from the states of NSW and QLD, respectively. The fourth column shows results for newspapers in regions where support for the Coalition party exceeded 50% in the 2016 elections. Results for the Low Coalition sample are omitted due to small sample size.*Note*: *** p<0.01, ** p<0.05, * p<0.1.

Sample	All States	NSW State	QLD State	High Coalition
Coefficient	-0.006	0.045	0.078	0.014
Std. Error	0.065	0.164	0.141	0.086
N. Obs	2,898	1,638	234	1710
Q1	0	-0.130	-0.076	-0.083
Mean	0.157	0.058	0.166	0.140
Q3	0.304	0.263	0.377	0.308
SD	0.364	0.285	0.378	0.408

**Fig 5:**
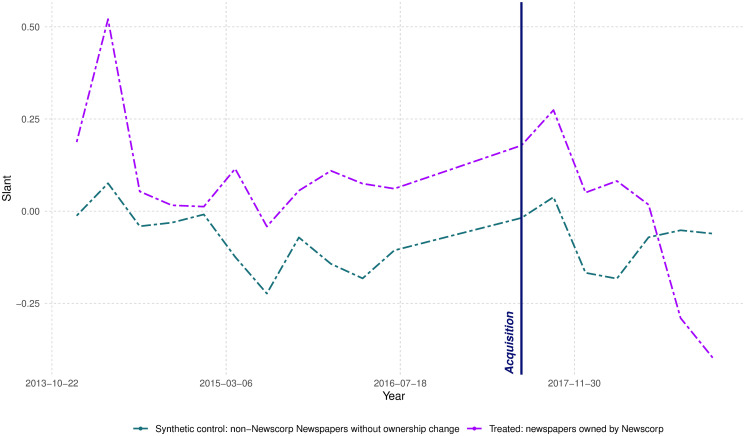
Change in political slant for existing News Corp newspapers. This figure illustrates the results of a Synthetic Difference-in-Differences (SDID) analysis. For this analysis, the control group consists of non-News Corp newspapers that remained under the same ownership, whereas the treatment group includes newspapers that always belonged to News Corp. The dot-dashed purple line represents the average trend of the treated group, while the dot-dashed green line shows the average time trend for the synthetic control. Solid purple and green lines depict the average linear trend between pre-treatment and post-treatment periods for the treatment and synthetic control groups, respectively. Small black error bars provide graphical estimates of the synthetic difference-in-differences effect. The vertical navy line indicates the first post-event observation.

News Corp owned several newspapers that did not change hands in 2016. We can also explore how their media slant changed after the acquisition. We repeat a similar analysis, but now our treatment group consists of newspapers that belonged to News Corp during all years in our sample. We construct a synthetic control group based on newspapers that did not change ownership. The results are reported in Fig 5. Here, we observe a strong relative shift toward a conservative slant among the newspapers that belonged to the conglomerate. Specifically, we find that the confidence interval for the change is [-0.228, -0.059], suggesting a strong shift toward a pro-Coalition slant. This result aligns with the conservative leaning of News Corp’s owner, Rupert Murdoch [64, 65]. The estimates are close to what [30] provide as the correlation between the estimated slant and the newspaper ideology (23.2% of observed slant).

Table 3 provides a more detailed analysis of the results. The first column indicates that, on average, existing newspapers changed their political leaning by 0.143 towards the Coalition party. This coefficient is highly significant and corresponds to 26% ( = 0.143/0.555) of standard deviation of pre-event slant for acquired newspapers. The second column focuses on the newspapers in the state of New South Wales and finds a similar result. We also find support for the change in political leaning of existing newspapers in the states of Queensland and Victoria. In the last two columns, we compare the electoral regions with high Coalition party support with those with low Coalition party support during the elections prior to the acquisition. We find that the strongest change in the political leaning of the newspapers is concentrated in the regions with higher Coalition party support prior to the acquisition, as indicated by the statistically significant difference between coefficients in the models, estimated with Z-score $(\left(-0.24-\left(-0.06\right)\right)/\left(\sqrt{0.064^{2}+0.062^{2}}\right)\approx -2.02$).

**Table 3:** Shows the change in political slant in News Corp newspaper. This table presents the estimates of a Synthetic Difference-in-Differences analysis that compares the trends of newspapers that belonged to News Corp prior to 2016 acquisitions and a synthetic control group. The first row provides the point estimate of the difference, the second row shows the “jackknife” estimated standard errors, and the third row shows the total number of newspaper-quarter observations for both the treatment and control samples combined. Rows four to seven present summary statistics for the treatment sample during the pre-event window. Specifically, the fourth (sixth) row reports the 25th (75th) percentile, the fifth row shows the mean, and the seventh row provides the standard deviation. The first column indicates the results for the full sample, the second (third/fourth/fifth) column focuses on the newspapers in the states of NSW (QLD/VIC/SA). The sixth (seventh) column shows the results for newspapers in the regions that had support for the Coalition party above (below) 50% in the 2016 elections.*Note*: *** p<0.01, ** p<0.05, * p<0.1.

Sample	All States	NSW States	QLD State	VIC State	SA State	Low Coalition	High Coalition
Coefficient	-0.143***	-0.194***	0.222***	-0.213***	0.109	-0.240***	-0.06
Std. Error	0.043	0.072	0.079	0.069	0.099	0.064	0.062
N. Obs	4,752	2,034	630	936	468	2,106	2,340
Q1	-0.222	-0.217	-0.434	0	-0.333	-0.082	-0.377
Mean	0.061	0.064	-0.121	0.292	-0.122	0.133	-0.009
Q3	0.351	0.335	0.222	0.667	0.111	0.399	0.333
SD	0.555	0.509	0.483	0.605	0.434	0.541	0.584

We report a similar analysis for the Nine/Fairfax merger and the acquisition of Australian Community Media by Catalano and Waislitz. Unlike News Corp, these companies were not owning major newspapers prior to the acquisitions in 2018 and 2019. Hence, we only focus on the acquired newspapers for these two acquisitions. First, we focus on the Nine Entertainment Acquisition, which changed the ownership of 118 newspapers in our sample, mostly in the states of New South Wales and Victoria. We repeat the SDID analysis following the previous sections, with the treatment group comprised of newspapers acquired by the conglomerate and the control pool consisting of newspapers that did not change ownership. We use the SDID analysis based on the quarterly estimates of political slant, focusing on the interval of three years before the acquisition and two years after the acquisition, excluding the quarter of the acquisition and the quarter after it. Table 4 shows the results of the SDID estimates. We find that the acquired newspapers shifted towards the Labor party and away from the Coalition party in their political slant. For the full sample, the estimate of the shift equals 0.197 and corresponds to 34% of standard deviation of political slant of acquired newspapers before the acquisition, with a 95% confidence interval of [0.120, 0.273]. Subsequent columns in the table show that newspapers in the states of New South Wales and Victoria both experienced a similar shift in bias. The last two columns indicate that the majority of the change in political slant is concentrated in the regions that showed higher Coalition support in the elections prior to the acquisition, but this difference is not statistically significant according to Z-score.

**Table 4:** Shows the change in political slant in newspapers acquired by Nine Entertainment in 2018. This table presents the estimates of a Synthetic Difference-in-Differences analysis that compares the trends of newspapers acquired by Nine Entertainment in 2018 with synthetic control. The first row provides the point estimate of the difference, the second row shows the “jackknife” estimated standard errors, and the third row shows the total number of newspaper-quarter observations for both the treatment and control samples combined. Rows four to seven present summary statistics for the treatment sample during the pre-event window. Specifically, the fourth (sixth) row reports the 25th (75th) percentile, the fifth row shows the mean, and the seventh row provides the standard deviation. The first column indicates the results for the full sample, the second column focuses on the newspapers in NSW, the third column on VIC, and the fourth and fifth columns show the results for newspapers in regions with high (>50%) and low (<50%) Coalition support, respectively.*Note*: *** p<0.01, ** p<0.05, * p<0.1.

Sample	All States	NSW State	VIC State	QLD State	Low Coalition	High Coalition
Coefficient	0.197***	0.277***	0.215***	-0.064	0.159**	0.223***
Std. Error	0.039	0.054	0.077	0.186	0.062	0.057
N. Obs	4,063	1,785	833	578	1,836	2,074
Q1	-0.412	-0.389	-0.003	-0.689	-0.2	-0.494
Mean	-0.05	-0.018	0.131	-0.374	0.097	-0.134
Q3	0.333	0.374	0.467	-0.129	0.448	0.204
SD	0.582	0.594	0.451	0.467	0.564	0.558

Lastly, we analyze the effect of the acquisition of Australian Community Media newspapers in 2019. We follow a similar methodology as before and report the results in Table 5. We find that the ACM acquisition of newspapers corresponded to an average increase of 0.089 in the pro-Labor slant of the newspapers compared to the synthetic control group. This number corresponds to 16% of the standard deviation for the sample of the acquired companies before the acquisition, and the 95% confidence interval equals [0.019, 0.159]. Table 5 also splits the results by states, and the stronger shift in political bias happens in the newspapers in the state of Victoria. Lastly, unlike all previous acquisitions, the ACM acquisitions have higher value of slant shift in low Coalition support regions but this difference is not statistically significant with the respect to Z-score test.

**Table 5:** Shows the change in political slant in newspapers belonging to Australian Community media and acquired by Anthony Catalano and Alex Waislitz. This table presents the estimates of a Synthetic Difference-in-Differences analysis that compares the trends of newspapers acquired by Catalano and Waislitz in 2019 with synthetic control. The first row provides the point estimate of the difference, the second row shows the “jackknife” estimated standard errors, and the third row shows the total number of newspaper-quarter observations for both the treatment and control samples combined. Rows four to seven present summary statistics for the treatment sample during the pre-event window. Specifically, the fourth (sixth) row reports the 25th (75th) percentile, the fifth row shows the mean, and the seventh row provides the standard deviation. The first column indicates the results for the full sample, the second column focuses on the newspapers in NSW, the third column on VIC, the fourth column on QLD, and the fifth and sixth columns show the results for newspapers in regions with high (>50%) and low (<50%) Coalition support, respectively.*Note*: *** p<0.01, ** p<0.05, * p<0.1.

Sample	All States	NSW State	VIC State	QLD State	Low Coalition	High Coalition
Coefficient	0.089**	0.036	0.259**	-0.013	0.132**	0.035
Std. Error	0.036	0.054	0.116	0.092	0.054	0.049
N. Obs	3978	1782	720	630	1782	2034
Q1	-0.438	-0.381	-0.204	-0.632	-0.333	-0.467
Mean	-0.085	-0.023	0.104	-0.331	0.017	-0.146
Q3	0.311	0.333	0.542	-0.002	0.389	0.220
SD	0.569	0.531	0.521	0.409	0.576	0.545

## Discussion

In conclusion, this paper presents novel evidence on how media acquisitions by corporate conglomerates can influence the political slant of newspapers. We developed and validated a measure of political slant across major Australian newspapers, finding that acquisitions by large media firms can shift the slant of acquired papers toward the presumed preferences of their new corporate owners. Notably, we also discovered evidence that these acquisitions could alter the slant of other newspapers already owned by the conglomerates, suggesting a coordinating influence on the overall ideological positioning of the corporate media portfolio.

These findings raise significant concerns over increasing media consolidation and polarization. The Australian case demonstrates that concentrated corporate ownership can enable powerful firms to broadly shape media rhetoric and narratives across their holdings. While our analysis focused on print media, future research could examine whether similar patterns emerge across other platforms such as online outlets, television, and radio. Another potential extension of this study is to explore more granular measures of political slant based on specific topics discussed in the news, such as economics or the environment. Both extensions would provide a deeper understanding of the dynamics between media ownership and political slant.

Our study addresses a gap in the literature by highlighting the role of supply rather than demand factors in media slant. These factors may become more prevalent due to modern trends such as the rise of media ownership concentration [14], the decline in local newspapers [22], and favorable conditions for media mergers [2]. This paper underscores the importance of diverse media ownership as a key policy implication for regulators.

## Supporting information

S1 TableRobustness: Regular difference-in-differnece analysis.This table estimates a standard difference-in-differences specification as a robustness check to the synthetic difference-in-differences analysis presented in the main body of the paper. Specifically, we follow the same steps to construct treatment and control groups, then estimate the following regression:
$$Slant_{n,t}=Treated_{n,t}\times Post_{n,t}+\kappa_{n}+\zeta_{t}+\varepsilon_{n,t}$$
In this specification, *κ*_*n*_ represents newspaper fixed effects, and *ζ*_*t*_ represents time fixed effects (FE), both of which absorb the individual treatment and post-period coefficients. Standard errors are clustered at the newspaper level to account for within-newspaper correlation.(PDF)

S2 Table Additional summary statistics. This table provides additional summary statistics for Tables 2–5. Specifically, the first four rows of each panel present the 25th percentile, mean, 75th percentile, and standard deviation for all observations in the treatment and pooled synthetic control groups across all (pre- and post-) periods. The last four rows present statistics for the treatment group only, covering both the pre- and post-event periods.
(PDF)

S3 Table Robustness check: Alternative trigram selection for political slant (Based on Table 2). This table re-estimates the analysis from Table 2, using a political slant measure based on a different number of trigrams, rather than the 150 trigrams used in the main analysis.
(PDF)

S4 Table Robustness check: Alternative trigram selection for political slant (Based on Table 3). This table re-estimates the analysis from Table 3, using a political slant measure based on a different number of trigrams, rather than the 150 trigrams used in the main analysis.
(PDF)

S5 Table Robustness check: Alternative trigram selection for political slant (Based on Table 4). This table re-estimates the analysis from Table 4, using a political slant measure based on a different number of trigrams, rather than the 150 trigrams used in the main analysis.
(PDF)

S6 Table Robustness check: Alternative trigram selection for political slant (Based on Table 5). This table re-estimates the analysis from Table 5, using a political slant measure based on a different number of trigrams, rather than the 150 trigrams used in the main analysis.
(PDF)

S7 Table Weights of the synthetic control group (Based on Table 2). This table reports the weights assigned to newspapers in the synthetic control group used for the analysis in Table 2.
(PDF)

S8 Table Weights of the synthetic control group (Based on Table 3). This table reports the weights assigned to newspapers in the synthetic control group used for the analysis in Table 3.
(PDF)

S9 Table Weights of the synthetic control group (Based on Table 4). This table reports the weights assigned to newspapers in the synthetic control group used for the analysis in Table 4.
(PDF)

S10 Table Weights of the synthetic control group (Based on Table 5). This table reports the weights assigned to newspapers in the synthetic control group used for the analysis in Table 5.
(PDF)

S1 Data (ZIP)
