# Supplementary material for: Media ownership and ideological slant: Evidence from Australian newspaper mergers
Source: PLoS One. 2024 Dec 31;19(12):e0315137. doi: 10.1371/journal.pone.0315137 (PMC11687783; doi:10.1371/journal.pone.0315137)
Supplement: S1 Table — This table estimates a standard difference-in-differences specification as a robustness check to the synthetic difference-in-differences analysis presented in the main body of the paper. Specifically, we follow the same steps to construct treatment and control groups, then estimate the following regression: Slantn,t=Treatedn,t×Postn,t+κn+ζt+εn,t In this specification, κn represents newspaper fixed effects, and ζt represents time fixed effects (FE), both of which absorb the individual treatment and post-period coefficients. Standard errors are clustered at the newspaper level to account for within-newspaper correlation. (PDF) [file pone.0315137.s001.pdf]

(a) Difference-in-Differences Analysis Based on Table 2

	All States	NSW State	QLD State	High Coalition
Treated × Post	-0.023	0.015	-0.014	-0.003
	(0.066)	(0.132)	(0.146)	(0.087)
Num.Obs.	2,898	1,638	234	1,710
R2	0.321	0.273	0.507	0.247
FE: newspaper	X	X	X	X
FE: quarter	X	X	X	X

(b) Difference-in-Differences Analysis Based on Table 3

	All States	NSW State	QLD State	SA State	High Coalition	Low Coalition
Treated × Post	-0.123***	-0.164**	0.128	0.166	-0.189***	-0.065
	(0.042)	(0.067)	(0.121)	(0.119)	(0.064)	(0.057)
Num.Obs.	4,752	2,034	630	468	2,106	2,340
R2	0.313	0.281	0.277	0.341	0.347	0.263
FE: newspaper	X	X	X	X	X	X
FE: quarter	X	X	X	X	X	X

(c) Difference-in-Differences Analysis Based on Table 4

	All States	NSW State	VIC State	QLD State	High Coalition	Low Coalition
Treated × Post	0.206***	0.254***	0.348***	0.093	0.229***	0.200***
	(0.039)	(0.057)	(0.076)	(0.064)	(0.058)	(0.055)
Num.Obs.	4,063	1,785	833	578	1,836	2,074
R2	0.344	0.330	0.398	0.372	0.356	0.312
FE: newspaper	X	X	X	X	X	X
FE: quarter	X	X	X	X	X	X

(d) Difference-in-Differences Analysis Based on Table 5

	(1) All States	(2) NSW State	(3) VIC State	(4) QLD State	(5) High Coalition	(6) Low Coalition
Treated × Post	0.192***	0.245***	0.310**	-0.037	0.237***	0.167***
	(0.039)	(0.058)	(0.118)	(0.064)	(0.061)	(0.054)
Num.Obs.	3,978	1,782	720	630	1,782	2,034
R2	0.319	0.290	0.402	0.332	0.333	0.295
FE: newspaper	X	X	X	X	X	X
FE: time	X	X	X	X	X	X
