# Supplementary material for: Media ownership and ideological slant: Evidence from Australian newspaper mergers
Source: PLoS One. 2024 Dec 31;19(12):e0315137. doi: 10.1371/journal.pone.0315137 (PMC11687783; doi:10.1371/journal.pone.0315137)
Supplement: S2 Table — This table provides additional summary statistics for Tables 2–5. Specifically, the first four rows of each panel present the 25th percentile, mean, 75th percentile, and standard deviation for all observations in the treatment and pooled synthetic control groups across all (pre- and post-) periods. The last four rows present statistics for the treatment group only, covering both the pre- and post-event periods. (PDF) [file pone.0315137.s002.pdf]

(a) Additional Summary Statistics for Table 2

	All States	NSW	QLD	High Coal
		**Full Sample**		
Q1	-0.345	-0.382	-0.319	-0.444
Mean	-0.059	-0.049	-0.036	-0.126
Q3	0.254	0.289	0.269	0.196
SD	0.550	0.565	0.477	0.551
		**Treated Subsample**		
Q1	-0.018	-0.112	-0.052	-0.083
Mean	0.132	0.072	0.178	0.132
Q3	0.281	0.274	0.382	0.320
SD	0.332	0.336	0.378	0.376

(b) Additional Summary Statistics for Table 3

	All States	NSW	QLD	VIC	SA	High Coal	Low Coal
				**Full Sample**			
Q1	-0.333	-0.333	-0.503	0	-0.577	-0.167	-0.448
Mean	-0.016	-0.029	-0.171	0.251	-0.246	0.094	-0.108
Q3	0.333	0.333	0.127	0.625	0.037	0.409	0.222
SD	0.564	0.564	0.493	0.583	0.536	0.542	0.573
				**Treated Subsample**			
Q1	-0.222	-0.222	-0.467	0	-0.330	-0.110	-0.372
Mean	0.060	0.055	-0.136	0.277	-0.084	0.121	-0.013
Q3	0.337	0.370	0.217	0.667	0.111	0.389	0.333
SD	0.554	0.526	0.497	0.607	0.416	0.533	0.583

(c) Additional Summary Statistics for Table 4

	All States	NSW	VIC	QLD	Low Coal	High Coal
			**Full Sample**			
Q1	-0.222	-0.295	0	-0.328	-0.030	-0.384
Mean	0.028	0.036	0.193	-0.020	0.121	-0.054
Q3	0.333	0.374	0.447	0.233	0.400	0.259
SD	0.521	0.559	0.438	0.490	0.490	0.540
			**Treated Subsample**			
Q1	-0.389	-0.359	-0.027	-0.672	-0.169	-0.500
Mean	-0.044	0.005	0.125	-0.38	0.099	-0.134
Q3	0.333	0.389	0.453	-0.105	0.455	0.208
SD	0.567	0.595	0.45	0.438	0.545	0.563

(d) Additional Summary Statistics for Table 5

	All States	NSW	VIC	QLD	Low Coal	High Coal
			**Full Sample**			
Q1	-0.249	-0.278	0	-0.322	-0.066	-0.388
Mean	-0.006	0.005	0.128	-0.037	0.078	-0.080
Q3	0.280	0.333	0.333	0.210	0.333	0.222
SD	0.509	0.522	0.454	0.476	0.482	0.523
			**Treated Subsample**			
Q1	-0.436	-0.380	-0.305	-0.617	-0.289	-0.500
Mean	-0.082	-0.025	0.069	-0.330	0.039	-0.154
Q3	0.307	0.333	0.501	-0.004	0.408	0.207
SD	0.566	0.555	0.537	0.443	0.574	0.550
