# Supplementary material for: Media ownership and ideological slant: Evidence from Australian newspaper mergers
Source: PLoS One. 2024 Dec 31;19(12):e0315137. doi: 10.1371/journal.pone.0315137 (PMC11687783; doi:10.1371/journal.pone.0315137)
Supplement: S3 Table — This table re-estimates the analysis from Table 2, using a political slant measure based on a different number of trigrams, rather than the 150 trigrams used in the main analysis. (PDF) [file pone.0315137.s003.pdf]

(a) Variation with 50 Trigrams

Sample	All States	NSW	QLD	High Co
Coefficient	0.007	-0.041	-0.003	0.012
Std. Error	0.035	0.103	0.08	0.056
N. Obs	2700	1638	234	1710

(b) Variation with 100 Trigrams

Sample	All States	NSW	QLD	High Co
Coefficient	0.028	0.056	0.146*	0.071
Std. Error	0.042	0.133	0.078	0.059
N. Obs	2700	1638	234	1710

(c) Variation with 200 Trigrams

Sample	All States	NSW	QLD	High Co
Coefficient	-0.006	-0.119	0.044	0.02
Std. Error	0.044	0.15	0.071	0.067
N. Obs	2700	1620	252	1710
