# Supplementary material for: Media ownership and ideological slant: Evidence from Australian newspaper mergers
Source: PLoS One. 2024 Dec 31;19(12):e0315137. doi: 10.1371/journal.pone.0315137 (PMC11687783; doi:10.1371/journal.pone.0315137)
Supplement: S4 Table — This table re-estimates the analysis from Table 3, using a political slant measure based on a different number of trigrams, rather than the 150 trigrams used in the main analysis. (PDF) [file pone.0315137.s004.pdf]

(a) Variation with 50 Trigrams

Sample	All	NSW	QLD	VIC	SA	Low Co	High Co
Coefficient	-0.064**	-0.037	0.07	-0.104***	0.259***	-0.108**	-0.026
Std. Error	0.032	0.047	0.077	0.038	0.079	0.042	0.049
N. Obs	4482	2016	630	936	468	2088	2340

(b) Variation with 100 Trigrams

Sample	All	NSW	QLD	VIC	SA	Low Co	High Co
Coefficient	-0.067*	-0.091	0.264***	-0.192***	0.202***	-0.18***	-0.012
Std. Error	0.035	0.056	0.067	0.044	0.077	0.049	0.047
N. Obs	4482	2016	630	936	468	2088	2340

(c) Variation with 200 Trigrams

Sample	All	NSW	QLD	VIC	SA	Low Co	High Co
Coefficient	-0.046	-0.042	0.161***	-0.343***	0.257***	-0.206***	0.093*
Std. Error	0.036	0.048	0.048	0.052	0.082	0.047	0.053
N. Obs	4518	2052	648	936	450	2088	2376
