# Supplementary material for: Media ownership and ideological slant: Evidence from Australian newspaper mergers
Source: PLoS One. 2024 Dec 31;19(12):e0315137. doi: 10.1371/journal.pone.0315137 (PMC11687783; doi:10.1371/journal.pone.0315137)
Supplement: S5 Table — This table re-estimates the analysis from Table 4, using a political slant measure based on a different number of trigrams, rather than the 150 trigrams used in the main analysis. (PDF) [file pone.0315137.s005.pdf]

(a) Variation with 50 Trigrams

Sample	All	NSW	VIC	QLD	Low Co	High Co
Coefficient	0.074**	0.065	-0.006	0	0.004	0.108***
Std. Error	0.032	0.042	0.058	0.236	0.048	0.04
N. Obs	3876	1751	816	578	1819	2006

(b) Variation with 100 Trigrams

Sample	All	NSW	VIC	QLD	Low Co	High Co
Coefficient	0.116***	0.151***	0.034	-0.021	0.081*	0.11***
Std. Error	0.029	0.049	0.037	0.166	0.047	0.038
N. Obs	3927	1785	816	578	1819	2057

(c) Variation with 200 Trigrams

Sample	All	NSW	VIC	QLD	Low Co	High Co
Coefficient	0.131***	0.139***	0.142**	-0.016	0.116***	0.156***
Std. Error	0.028	0.037	0.068	0.128	0.037	0.042
N. Obs	4029	1853	833	578	1870	2108
