# Supplementary material for: Media ownership and ideological slant: Evidence from Australian newspaper mergers
Source: PLoS One. 2024 Dec 31;19(12):e0315137. doi: 10.1371/journal.pone.0315137 (PMC11687783; doi:10.1371/journal.pone.0315137)
Supplement: S6 Table — This table re-estimates the analysis from Table 5, using a political slant measure based on a different number of trigrams, rather than the 150 trigrams used in the main analysis. (PDF) [file pone.0315137.s006.pdf]

(a) Variation with 50 Trigrams

Sample	All	NSW	VIC	QLD	Low Co	High Co
Coefficient	0.057*	-0.095***	0.083	0.028	0.003	0.057
Std. Error	0.034	0.031	0.091	0.121	0.053	0.042
N. Obs	3621	1666	680	595	1666	1904

(b) Variation with 100 Trigrams

Sample	All	NSW	VIC	QLD	Low Co	High Co
Coefficient	0.07**	0.053	0.006	-0.103	0.022	0.082**
Std. Error	0.028	0.044	0.138	0.109	0.041	0.041
N. Obs	3638	1666	697	595	1666	1921

(c) Variation with 200 Trigrams

Sample	All	NSW	VIC	QLD	Low Co	High Co
Coefficient	0.076***	0.096**	0.171	-0.018	0.116**	0.053
Std. Error	0.028	0.041	0.15	0.068	0.048	0.039
N. Obs	3672	1700	697	595	1666	1955
