# Supplementary material for: Media ownership and ideological slant: Evidence from Australian newspaper mergers
Source: PLoS One. 2024 Dec 31;19(12):e0315137. doi: 10.1371/journal.pone.0315137 (PMC11687783; doi:10.1371/journal.pone.0315137)
Supplement: S7 Table — This table reports the weights assigned to newspapers in the synthetic control group used for the analysis in Table 2. (PDF) [file pone.0315137.s007.pdf]

Newspaper	Weight	Newspaper	Weight
Merimbula News Weekly	0.0101	Blacktown City Sun	0.0069
The Northern Argus	0.0098	The Northern Daily Leader	0.0068
Port Lincoln Times	0.0097	The North Queensland Register	0.0068
Maribyrnong & Hobsons Bay Weekly	0.0093	The Narromine News	0.0067
Singleton Argus	0.0093	Southern Cross	0.0067
Merredin – Wheatbelt Mercury	0.0092	Narooma News	0.0067
Donnybrook – Bridgetown Mail	0.0090	The Rural	0.0067
The Camden Haven Courier	0.0090	The Standard	0.0067
Wauchope Gazette	0.0090	Wingham Chronicle	0.0066
Hills News	0.0088	The Islander	0.0066
Moree Champion	0.0087	The Flinders News	0.0066
Bega District News	0.0087	Central Midlands and Coastal Advocate	0.0066
Maitland Mercury	0.0087	Eastern Riverina Chronicle	0.0066
The Manning River Times	0.0085	Wyndham Weekly	0.0066
Augusta – Margaret River Mail	0.0084	The Boorowa News	0.0066
The Irrigator	0.0084	The Transcontinental	0.0065
Redland City Bulletin	0.0084	Southern Weekly	0.0065
Magnet	0.0083	Liverpool City Champion	0.0065
Stock and Land	0.0083	Stock Journal	0.0065
Milton Ulladulla Times	0.0082	The Age	0.0065
Bunbury Mail	0.0082	Kiama Independent	0.0064
Northern Weekly	0.0082	Illawarra Mercury	0.0064
The Inverell Times	0.0080	The Murray Valley Standard	0.0064
Melton & Moorabool Weekly	0.0080	Cootamundra Herald	0.0064
The Naracoorte Herald	0.0080	The Grenfell Record and Bland Advertiser	0.0064
Port Macquarie News	0.0080	Western Advocate	0.0064
Sunbury & Macedon Ranges Weekly	0.0079	Daily Liberal and Macquarie Advocate	0.0064
Glen Innes Examiner	0.0079	Eyre's Peninsula Tribune	0.0063
The Recorder	0.0079	The Land	0.0063
Hawkesbury Gazette	0.0078	The Canberra Times	0.0062
Crookwell Gazette	0.0077	Coastal Leader	0.0062
The Young Witness	0.0077	Town & Country Magazine	0.0062
The Dungog Chronicle	0.0076	Penrith City Gazette	0.0062
The Tenterfield Star	0.0075	Mandurah Mail	0.0062
Goulburn Post	0.0075	The Collie Mail	0.0062
The Examiner	0.0075	Sunday Age	0.0061
St. George and Sutherland Shire Leader	0.0075	Herald	0.0061
The Avon Valley Advocate	0.0075	Western Magazine	0.0061
Northern News	0.0075	Braidwood Times	0.0061
Blayney Chronicle	0.0075	Lithgow Mercury	0.0061
Queensland Country Life	0.0075	The Bellingen Shire Courier – Sun	0.0060
The Sun (Parramatta, Holroyd)	0.0074	The Border Chronicle	0.0060
Southern Highland News	0.0074	The Advertiser	0.0060
The North West Star	0.0074	Nyngan Observer	0.0060
The Queanbeyan Age	0.0074	The Cowra Guardian	0.0059
Port Stephens Examiner	0.0074	Roxby Downs Sun	0.0059
Whyalla News	0.0074	The Bendigo Advertiser	0.0058
Brimbank & North West Weekly	0.0074	Bombala Times	0.0058
The Esperance Express	0.0073	The Times	0.0057
Advocate	0.0073	Blue Mountains Gazette	0.0057
The Harden – Murrumburrah Express	0.0073	The Wimmera Mail – Times	0.0057
South West Advertiser	0.0073	Great Lakes Advocate	0.0056
Advocate	0.0072	Wellington Times	0.0056
The Advertiser	0.0072	Oberon Review	0.0055
The Courier	0.0072	The Goondiwindi Argus	0.0055
The Scone Advocate	0.0072	Yass Tribune	0.0054
The Daily Advertiser	0.0071	The Walcha News	0.0053
Katherine Times	0.0071	Bay Post	0.0053
Bayside Bulletin	0.0071	Ararat Advertiser and Stawell Times	0.0053
Central Western Daily	0.0071	Area News	0.0053
The Armidale Express	0.0071	The Border Mail	0.0052
Fairfield City Champion	0.0071	Guyra Argus	0.0050
Busselton – Dunsborough Mail	0.0071	The Star	0.0049
Sydney Morning Herald	0.0070	Cooma Monaro Express	0.0048
Forbes Advocate	0.0070	Macleay Argus	0.0048
South Coast Register	0.0070	Canowindra News	0.0047
St. Marys Star	0.0070	Parkes Champion Post	0.0047
The Gloucester Advocate	0.0070	Wollondilly Advertiser	0.0045
The West Coast Sentinel	0.0070	The Ridge News	0.0044
Farm Weekly	0.0069	Mudgee Guardian and Gulgong Advertiser	0.0042
Sun Herald	0.0069	Campbelltown – Macarthur Advertiser	0.0042
The Muswellbrook Chronicle	0.0069	Camden Advertiser	0.0042
Guardian News	0.0069		
