# Supplementary material for: Media ownership and ideological slant: Evidence from Australian newspaper mergers
Source: PLoS One. 2024 Dec 31;19(12):e0315137. doi: 10.1371/journal.pone.0315137 (PMC11687783; doi:10.1371/journal.pone.0315137)
Supplement: S8 Table — This table reports the weights assigned to newspapers in the synthetic control group used for the analysis in Table 3. (PDF) [file pone.0315137.s008.pdf]

Newspaper	Weight	Newspaper	Weight
Goulburn Post	0.0102	The Walcha News	0.0069
Northern News	0.0099	Farm Weekly	0.0068
Bombala Times	0.0098	Melton & Moorabool Weekly	0.0068
Sunday Age	0.0096	The Bellingen Shire Courier - Sun	0.0068
The Islander	0.0096	St. Marys Star	0.0068
The Queanbeyan Age	0.0096	The Boorowa News	0.0067
Merimbula News Weekly	0.0094	The North West Star	0.0067
The Irrigator	0.0094	Blue Mountains Gazette	0.0067
The Northern Argus	0.0093	Cootamundra Herald	0.0067
The Muswellbrook Chronicle	0.0093	Guardian News	0.0066
Parkes Champion Post	0.0092	The Northern Daily Leader	0.0066
The Armidale Express	0.0092	The Canberra Times	0.0066
Sun Herald	0.0092	The Grenfell Record and Bland Advertiser	0.0066
Northern Weekly	0.0090	Town & Country Magazine	0.0066
Central Western Daily	0.0090	Bayside Bulletin	0.0066
Cooma Monaro Express	0.0088	Yass Tribune	0.0066
The Camden Haven Courier	0.0088	Oberon Review	0.0066
Great Lakes Advocate	0.0088	Advocate	0.0066
Fairfield City Champion	0.0088	The Rural	0.0065
South Coast Register	0.0087	Lithgow Mercury	0.0065
The Manning River Times	0.0086	Wollondilly Advertiser	0.0065
Daily Liberal and Macquarie Advocate	0.0085	Sydney Morning Herald	0.0065
Hills News	0.0083	The Border Chronicle	0.0064
Port Lincoln Times	0.0082	Central Midlands and Coastal Advocate	0.0064
The Naracoorte Herald	0.0081	The Tenterfield Star	0.0064
The Sun (Parramatta, Holroyd)	0.0081	The Narromine News	0.0064
The Dungog Chronicle	0.0080	The Gloucester Advocate	0.0064
The Murray Valley Standard	0.0080	Guyra Argus	0.0063
Redland City Bulletin	0.0079	Maitland Mercury	0.0062
Wingham Chronicle	0.0079	The Standard	0.0062
Blayney Chronicle	0.0079	Eyre's Peninsula Tribune	0.0062
Eastern Riverina Chronicle	0.0079	Campbelltown - Macarthur Advertiser	0.0062
The Daily Advertiser	0.0079	Brimbank & North West Weekly	0.0062
St. George and Sutherland Shire Leader	0.0078	Canowindra News	0.0061
Busselton - Dunsborough Mail	0.0078	The Goondiwindi Argus	0.0061
Forbes Advocate	0.0077	The Esperance Express	0.0061
The Collie Mail	0.0077	Area News	0.0059
Stock Journal	0.0077	Wyndham Weekly	0.0059
Blacktown City Sun	0.0077	The Star	0.0059
Queensland Country Life	0.0077	Herald	0.0058
Port Stephens Examiner	0.0076	The Bendigo Advertiser	0.0058
Donnybrook - Bridgetown Mail	0.0076	Bunbury Mail	0.0057
Southern Cross	0.0076	Mudgee Guardian and Gulgong Advertiser	0.0057
Katherine Times	0.0076	Mandurah Mail	0.0057
Southern Weekly	0.0076	The Transcontinental	0.0057
The Harden - Murrumburrah Express	0.0075	Macleay Argus	0.0057
Glen Innes Examiner	0.0075	Nyngan Observer	0.0057
Merredin - Wheatbelt Mercury	0.0075	Magnet	0.0056
Crookwell Gazette	0.0074	Whyalla News	0.0056
The Flinders News	0.0074	Milton Ulladulla Times	0.0056
The Recorder	0.0074	Narooma News	0.0055
The Cowra Guardian	0.0073	The Avon Valley Advocate	0.0054
The North Queensland Register	0.0073	Ararat Advertiser and Stawell Times	0.0054
Singleton Argus	0.0072	Bega District News	0.0052
Herald	0.0072	The Scone Advocate	0.0052
The Inverell Times	0.0072	Maribyrnong & Hobsons Bay Weekly	0.0051
The Times	0.0071	The Border Mail	0.0051
Advocate	0.0071	Southern Highland News	0.0050
The Advertiser	0.0071	Western Advocate	0.0050
The Courier	0.0071	The Ridge News	0.0050
The Advertiser	0.0071	Wauchope Gazette	0.0047
Illawarra Mercury	0.0071	The Wimmera Mail - Times	0.0047
The Age	0.0070	Camden Advertiser	0.0045
South West Advertiser	0.0070	Port Macquarie News	0.0043
Liverpool City Champion	0.0070	Kiama Independent	0.0040
Penrith City Gazette	0.0070	Wellington Times	0.0039
The Land	0.0069	Roxby Downs Sun	0.0038
Augusta - Margaret River Mail	0.0069	Moree Champion	0.0037
Stock and Land	0.0069	The Young Witness	0.0034
Hawkesbury Gazette	0.0069	The West Coast Sentinel	0.0034
Western Magazine	0.0069	Sunbury & Macedon Ranges Weekly	0.0034
Braidwood Times	0.0069	Coastal Leader	0.0034
The Examiner	0.0069	Bay Post	0.0034
