# Supplementary material for: Media ownership and ideological slant: Evidence from Australian newspaper mergers
Source: PLoS One. 2024 Dec 31;19(12):e0315137. doi: 10.1371/journal.pone.0315137 (PMC11687783; doi:10.1371/journal.pone.0315137)
Supplement: S9 Table — This table reports the weights assigned to newspapers in the synthetic control group used for the analysis in Table 4. (PDF) [file pone.0315137.s009.pdf]

Newspaper	Weight	Newspaper	Weight
Fairfield Advance	0.0114	Darwin Palmerston Sun	0.0082
The Albert and Logan News	0.0111	Echo	0.0082
Central Coast Express Advocate	0.0108	Southern Courier	0.0081
Brisbane News	0.0101	Maroondah Leader	0.0081
The Manly Daily	0.0101	Mornington Peninsula Leader	0.0081
Penrith Press	0.0099	The Cairns Post	0.0081
Rouse Hill Times	0.0099	Mt. Druitt - St. Mary's Standard	0.0081
Leader Messenger	0.0098	Greater Dandenong Leader	0.0081
Southern Weekly	0.0097	The Southern Star	0.0081
Bribie Weekly	0.0097	Midland Kalamunda Reporter	0.0080
Parramatta Advertiser	0.0096	Free Press Leader	0.0080
Tablelands Advertiser	0.0095	Moonee Valley Leader	0.0080
Blacktown Advocate	0.0095	The Herbert River Express	0.0080
City Messenger	0.0094	Wyndham Leader	0.0080
Inner - West Weekly	0.0093	Wynnum Herald	0.0080
North Shore Times	0.0093	Whittlesea Leader	0.0079
The Hills Shire Times	0.0093	Eastern Courier Messenger	0.0079
The Weekend Australian Magazine	0.0092	Townsville Sun	0.0079
Tasmanian Country	0.0092	Sunday Tasmanian	0.0079
Moreland Leader	0.0092	City North Messenger	0.0079
The Atherton Tablelander	0.0091	Melton Leader	0.0079
Mosman Daily	0.0090	Cooma Monaro Express	0.0079
The Leader	0.0090	The Northern Territory News	0.0079
Townsville Bulletin	0.0090	Geelong Advertiser	0.0079
Guardian Messenger	0.0090	Penrith City Gazette	0.0078
The Courier - Mail	0.0088	Progress Leader	0.0077
Wish	0.0088	Caboolture Shire Herald	0.0077
The Australian	0.0088	Weekly Times Messenger	0.0077
Weekend Australian	0.0088	The Daily Telegraph	0.0077
Sportsman	0.0087	Comment News	0.0076
City North News	0.0087	The Express	0.0076
The Weekly Times	0.0087	East Torrens Messenger	0.0076
The Mercury	0.0087	Portside Messenger	0.0076
Blacktown City Sun	0.0087	Wentworth Courier	0.0075
Northern District Times	0.0087	Innisfail Advocate	0.0075
Hills News	0.0086	Gold Coast Sun	0.0075
The Gold Coast Bulletin	0.0086	Melbourne Yarra Leader	0.0075
North Coast Times	0.0086	Moorabbin Glen Eira Kingston Leader	0.0075
North - West News	0.0086	Hobsons Bay Leader	0.0075
Bayside Leader	0.0086	Bowen Independent	0.0075
Macarthur Chronicle	0.0085	Heidelberg Leader	0.0074
Central	0.0085	Mordialloc - Chelsea Leader	0.0074
Pine Rivers Press	0.0085	Hills Gazette	0.0073
Hornsby and Upper North Shore Advocate	0.0085	Monash Leader	0.0073
The Redcliffe & Bayside Herald	0.0085	Northcote Leader	0.0073
Geelong News	0.0085	Whitehorse Leader	0.0072
Port Douglas & Mossman Gazette	0.0085	Northern Miner	0.0072
Southern Times Messenger	0.0085	City South News	0.0072
Herald Sun	0.0085	Maribyrnong Leader	0.0072
Sunday Telegraph	0.0085	Brimbank Leader	0.0072
The Sun (Parramatta, Holroyd)	0.0084	Eastern Riverina Chronicle	0.0070
Inner West Courier	0.0084	Knox Leader	0.0070
Hume Leader	0.0084	Stonnington Leader	0.0067
Sunbury Macedon Ranges Leader	0.0084	South - East Advertiser	0.0067
Berwick / Pakenham Cardinia Leader	0.0083	Westside News	0.0066
Manningham Leader	0.0083	The Cairns Sun	0.0066
Frankston Standard Leader / Hastings Leader	0.0083	Preston Leader	0.0066
South West News	0.0083	Lilydale Yarra Valley Leader	0.0064
News Mail Bundaberg	0.0083	The Burdekin Advocate	0.0061
St. Marys Star	0.0083	Cranbourne Leader	0.0059
The Centralian Advocate	0.0082		
