# Supplementary material for: Media ownership and ideological slant: Evidence from Australian newspaper mergers
Source: PLoS One. 2024 Dec 31;19(12):e0315137. doi: 10.1371/journal.pone.0315137 (PMC11687783; doi:10.1371/journal.pone.0315137)
Supplement: S10 Table — This table reports the weights assigned to newspapers in the synthetic control group used for the analysis in Table 5. (PDF) [file pone.0315137.s010.pdf]

Newspaper	Weight	Newspaper	Weight
The Atherton Tablelander	0.0143	Portside Messenger	0.0087
Echo	0.0128	Central Coast Express Advocate	0.0086
North Coast Times	0.0127	Southern Courier	0.0086
Southern Weekly	0.0126	City North Messenger	0.0085
Pine Rivers Press	0.0124	St. Marys Star	0.0083
Tablelands Advertiser	0.0123	East Torrens Messenger	0.0083
The Albert and Logan News	0.0118	The Burdekin Advocate	0.0082
Comment News	0.0118	Mosman Daily	0.0082
The Cairns Post	0.0116	The Northern Territory News	0.0081
The Weekend Australian Magazine	0.0115	Hills Gazette	0.0081
The Gold Coast Bulletin	0.0115	South West News	0.0081
Hume Leader	0.0114	The Express	0.0081
Caboolture Shire Herald	0.0114	Inner West Courier	0.0081
The Weekly Times	0.0114	Gold Coast Sun	0.0080
Weekend Australian	0.0113	Sunday Telegraph	0.0080
Bribie Weekly	0.0112	Moreland Leader	0.0079
News Mail Bundaberg	0.0111	Wentworth Courier	0.0078
Farm Weekly	0.0110	Weekly Times Messenger	0.0077
Sunday Tasmanian	0.0109	North - West News	0.0077
Hills News	0.0107	The Manly Daily	0.0076
Tasmanian Country	0.0106	Progress Leader	0.0075
Brisbane News	0.0105	Moonee Valley Leader	0.0075
Northern Miner	0.0104	Sunbury Macedon Ranges Leader	0.0074
The Daily Telegraph	0.0104	Greater Dandenong Leader	0.0074
Townsville Bulletin	0.0104	Southern Times Messenger	0.0073
Berwick / Pakenham Cardinia Leader	0.0102	Inner - West Weekly	0.0073
The Australian	0.0101	Eastern Courier Messenger	0.0073
Bowen Independent	0.0101	The Cairns Sun	0.0072
Sportsman	0.0100	The Herbert River Express	0.0071
Leader Messenger	0.0100	Rouse Hill Times	0.0067
Herald Sun	0.0100	The Leader	0.0066
Geelong News	0.0099	Fairfield Advance	0.0066
Blacktown Advocate	0.0099	South - East Advertiser	0.0064
The Redcliffe & Bayside Herald	0.0098	Moorabbin Glen Eira Kingston Leader	0.0061
Guardian Messenger	0.0098	Darwin Palmerston Sun	0.0059
The Mercury	0.0097	Knox Leader	0.0059
The Sun (Parramatta, Holroyd)	0.0097	Westside News	0.0058
The Centralian Advocate	0.0095	Bayside Leader	0.0058
Mornington Peninsula Leader	0.0094	The Hills Shire Times	0.0057
Melbourne Yarra Leader	0.0094	Macarthur Chronicle	0.0057
The Southern Star	0.0094	North Shore Times	0.0055
Port Douglas & Mossman Gazette	0.0094	Penrith Press	0.0052
Hobsons Bay Leader	0.0093	Whittlesea Leader	0.0052
Frankston Standard Leader / Hastings Leader	0.0092	Central	0.0052
Wyndham Leader	0.0092	Whitehorse Leader	0.0050
Brimbank Leader	0.0092	Heidelberg Leader	0.0050
Geelong Advertiser	0.0092	City North News	0.0049
Townsville Sun	0.0091	Maribyrnong Leader	0.0047
Blacktown City Sun	0.0091	Monash Leader	0.0045
Melton Leader	0.0090	Preston Leader	0.0044
The Courier - Mail	0.0090	Northcote Leader	0.0042
Penrith City Gazette	0.0090	Mordialloc - Chelsea Leader	0.0041
Wynnum Herald	0.0090	Stonnington Leader	0.0041
Mt. Druitt - St. Mary's Standard	0.0090	Northern District Times	0.0041
Innisfail Advocate	0.0090	Cranbourne Leader	0.0037
Midland Kalamunda Reporter	0.0089	Lilydale Yarra Valley Leader	0.0036
Eastern Riverina Chronicle	0.0089	Maroondah Leader	0.0035
Wish	0.0089	Manningham Leader	0.0035
Parramatta Advertiser	0.0088	Hornsby and Upper North Shore Advocate	0.0035
City Messenger	0.0087	City South News	0.0035
