# Supplementary material for: Media ownership and ideological slant: Evidence from Australian newspaper mergers
Source: PLoS One. 2024 Dec 31;19(12):e0315137. doi: 10.1371/journal.pone.0315137 (PMC11687783; doi:10.1371/journal.pone.0315137)
Supplement: S1 Data — (ZIP) [file pone.0315137.s011.zip › Data_Package/Figures.html]

Figures and Tables


# Figures and Tables

#### 2024-10-01

# Table 1

| 2000-2007 Period | 2008-2013 Period | 2013-2022 Period |
| --- | --- | --- |
| pharmaceut benefit scheme | coal seam ga | make end meet |
| make end meet | build educ revolut | action climat chang |
| paid matern leav | action climat chang | impact climat chang |
| credit card debt | occup health safeti | struggl make end |
| ratifi kyoto protocol | tackl climat chang | tackl climat chang |
| struggl make end | renew energi target | put food tabl |
| pressur interest rate | rise sea level | racial discrimin act |
| tackl climat chang | sea level rise | clean energi financ |
| full sale telstra | impact climat chang | invest renew energi |
| rise sea level | seam ga mine | famili domest violenc |
| effect margin tax | effect climat chang | live poverti line |
| medicar safeti net | extrem weather event | uluru statement heart |
| privat health fund | clean energi futur | support marriag equal |
| health care system | trade train centr | climat chang energi |
| nuclear wast dump | carbon captur storag | act climat chang |
| industri relat legisl | issu climat chang | tax cut big |
| pharmaceut benefit advisori | tax benefit part | penalti rate cut |
| get balanc right | cut compani tax | renew energi sector |
| relat small busi | commun infrastructur program | clean energi target |
| health insur premium | rental afford scheme | public servic job |

**Most Common Coalition Phrases**

| 2000-2007 Period | 2008-2013 Period | 2013-2022 Period |
| --- | --- | --- |
| unfair dismiss law | outlaw motorcycl gang | small medium busi |
| embryon stem cell | big new tax | mental health suicid |
| loan interest rate | man woman child | australian small busi |
| strong econom growth | everi man woman | black spot program |
| adult stem cell | privat health cover | small busi famili |
| home loan interest | econom fiscal outlook | mobil black spot |
| new job creat | electr price rise | cut red tape |
| protect nuclear safeti | cost carbon tax | health suicid prevent |
| radiat protect nuclear | health insur premium | busi famili enterpris |
| australian radiat protect | busi consum confid | small mediums busi |
| unfair dismiss claim | carbon tax cost | australian energi regul |
| natur heritag trust | carbon tax mine | small famili busi |
| good econom manag | increas electr price | creat local job |
| australian protect servic | new carbon tax | new job creat |
| state govern respons | carbon tax go | instant asset writeoff |
| state govern fail | australian health insur | reduc red tape |
| higher educ reform | health insur associ | small busi tax |
| famili law act | carbon tax increas | small busi sector |
| nation water initi | small busi sector | develop northern australia |
| cathol independ school | promis carbon tax | industri innov scienc |

# Figure 1

# Figure 2

# Figure 3

[1]
“” Cor with labor: 0.5, cor with coalition: -0.66, cor with total:
0.72

# Figure 4

# Figure 5
