# Supplementary material for: Media ownership and ideological slant: Evidence from Australian newspaper mergers
Source: PLoS One. 2024 Dec 31;19(12):e0315137. doi: 10.1371/journal.pone.0315137 (PMC11687783; doi:10.1371/journal.pone.0315137)
Supplement: Attachment — Submitted filename: Reponses to Referees.pdf [file pone.0315137.s012.pdf]

Dear Editor and Referees,

We are grateful for the opportunity to revise our paper for PLOS ONE. Hereby, we provide the summary of the revisions we implemented as well as the point-by-point responses to every issue raised by the referees. We believe that the paper became stronger and more readable as a result of the revision.

Summary of major changes:

- We have included more papers in the literature review (included those kindly suggested by the referees) and discussed the differences of our approach,
- We have included a summary of what has been reported about political leanings of Australia's media owners involved in the M&A deals analysed in the paper.
- We have cleaned up the main figures and provided more information in the results table to enhance readability and facilitate meaningful assessment of magnitude of estimates.
- We included robustness checks that estimate the effect of M&A deals on the slant of affected newspapers using traditional difference-in-difference methods and different selection of the number of trigrams in synthetic difference-in-difference method.
- We provided an additional discussion on the validity of our estimates of slant in the dynamic perspective.

We are looking forward to hearing your decision regarding our revised manuscript.

Cheers.

Dr Maxim Ananyev
A/Prof Ekaterina Volkova

Reviewer #1:

Comment 1
I understand that the publication criteria at PLOS ONE do not require the authors to comprehensively review the literature, and the contribution of a paper to the literature is irrelevant. However, not citing an earlier study that does essentially the same but for a different country is unethical and would constitute plagiarism. Garz and Rickardsson (2023 "Ownership and media slant: Evidence from Swedish newspapers", Kyklos; as a working paper available online since 2021) nearly has the same title, makes the same theoretical arguments, and uses the same method of text analysis. At a minimum, I recommend to reference the earlier paper, not least to avoid concerns about unethical behavior and plagiarism. Ideally, however, it would be nice to see a short discussion of how the submitted paper differs and how it adds to previous findings. For example, I think adding further evidence from another context (Australia) may help to generalize certain results; the focus on newspaper acquisitions is different; and using the synthetic difference-in-differences approach to study these mergers is novel and appealing.

We thank the referee for mentioning this highly relevant paper. We have included the following discussion on page 3:

> Another paper close to ours is Garz and Rickardsson (2023) which also measures slant by comparing the newspaper content with the speeches of politicians. Focusing on the Swedish newspapers, it finds that the slants of newspapers owned by the same firm correlate strongly even if they circulate on markets with different ideological preferences of the audience. Our paper, focusing on Australia's local newspapers, explores how the slant changes dynamically in response to the M&A activity of the media owners.

Comment 2.
More generally speaking, even though it might not be relevant for PLOS ONE, it seems that the paper would benefit from a proper literature review. While the reference list includes multiple up-to-date references to relevant non-scholarly work (e.g., Gallup polls or newspaper reports about acquisitions), with the exception of Bursztyn et al. (2023), the paper misses scholarly studies published during the past decade.

We greatly appreciate the referee's suggestion regarding the literature review. While it is true that PLOS ONE submission guidelines indicate that only a "brief review" of key literature is required in the Introduction, we agree that incorporating a more comprehensive review would enhance the paper. In response, we have revised the manuscript to include the following text on page 3:

> The study of the origins of media slant is of considerable academic interest. With the news consumption becoming more segregated (Gonzales-Bailon at el, 2023) and well-documented persuasive effects of media (Martin and Yurukoglu, 2017; Djourelova, 2023) understanding of the demand and supply of bias is crucial for understanding political and economic behaviour. Apart from the previously discussed Gentzkow and Shapiro (2010) and Garz and Rickardsson (2023), the

documented factors of media slant were political connections of management and owners (Guo et al, 2021), international trade negotiations (Wu, 2021), influence of network cable news on the local news (Widmer 2021), and ideology of the newsroom (Hassel et al, 2022).

In conclusion, I thank the authors for taking on this interesting project and wish them success moving forward with the paper.

We thank the referee for the thoughtful remarks.

Reviewer #2:

This is a solid paper. The topic is certainly important and the data analysis is impressive. The main results are plausible and interesting. There are a few issues which is why I answer "partly" re analysis being sound. Here are my main comment/suggestions:

Comment 1

-I don't know how well the paper addresses the question of whether bias is supply or demand driven, as claimed. Are the supply-side biases/leanings of the owners other than News Corps even discussed? Even their ideology is discussed very briefly, in just one sentence I think. I would discuss evidence or lack thereof of bias/ideology of each owner/ownership group, including owners who gave up ownership in each merger, in some earlier section before the results, perhaps the section describing the mergers.

We thank the referee for raising this important concern. We have added the following discussion on page 10

> News Corp is the largest owner of print media in Australia. Its proprietor and executive chairman (2013-2023) Rupert Murdoch is described as "staunch conservative"[1] and the outlets associated with him are described to exhibit a leaning towards a conservative side of the political spectrum (Martin and Yurukoglu, 2017).
>
> Fairfax Media was one of the oldest media companies in Australia. In 2012, after the Fairfax family sold its stake,[2] Gina Rinehart, Australian mining magnate known for her support for conservative politicians[3] and opposition to carbon tax,[4] became its largest shareholder. She sold her Fairfax stake in 2015.[5] Fairfax Media itself was not named as providing funds to political parties since 2012.
>
> Nine Entertainment is a publicly traded company with a diverse set of shareholders. According to Australian Electoral Commission, in 2012-2019, Nine Entertainment was named as providing funds to Australian Labor Party (more than 50,000 AUD over 7 years).[6] It should be also noted Fairfax journalists had adopted a charter asserting their independence.[7] It was also reported that the charter remained unsigned by Nine executives.[8]
>
> Australian Community Media is a media company responsible for a set of regional newspapers. It has been a part of Fairfax Media since 2007, then a part of Nine Entertainment (since the Nine/Fairfax merger in 2018). In 2018, it was bought and Antony Catalano and Alex Waislitz.[9]
>
> The new owners of Australian Community Media, Antony Catalano and Alex Waislitz, refrain from publicly expressing their political views, emphasising instead the business aspects in their appearances and interviews. Notably, Alex Waislitz's philanthropic foundation has partnered with the Clontarf

[1] https://www.politico.eu/article/rupert-murdoch-dominated-uk-politics-last-his-kind-fox-news-coproration-media/
[2] https://www.smh.com.au/business/fairfax-family-sells-out-of-company-20111110-1n96f.html
[3] https://www.ginarinehart.com.au/gina-rinehart-at-the-conservative-political-action-conference-in-maryland-on-saturday/
[4] https://www.theguardian.com/environment/blog/2011/may/31/mining-media-australia-carbon-tax
[5] https://www.smh.com.au/business/companies/gina-rinehart-sells-out-of-fairfax-media-20150206-138b5i.html
[6] https://transparency.aec.gov.au/ClientEntity/EntityDetail?clientIdentifier=21242&eventId=&clientType=organisationdonor
[7] https://www.smh.com.au/national/fairfax-media-charter-of-editorial-independence-20120619-20l4t.html
[8] https://theklaxon.com.au/home-nine-fairfax-independence-charter-a-sham-ecscp/
[9] https://www.abc.net.au/news/2019-04-30/nine-sells-fairfax-community-newspapers-to-the-cat/11058066

Foundation, an organisation dedicated to enhancing opportunities for Indigenous children.[10] It is also important to acknowledge that phrases surrounding Indigenous issues are associated with the speeches of Labor Party politicians. Neither Catalano nor Waislitz are shown on the returns of the Australian Electoral Commission as donors around the time of the acquisition.

I also think a crucial variable is ignored in the analysis, the degree of competition each newspaper faces in its metropolitan area. Supply-side bias is much more likely to influence slant in a paper with a local monopoly. It would be ideal to see the paper account for this factor but I'm not sure if it's necessary for publication in this journal. (I don't insist on this for this journal.)

We agree that this is an important question. Unfortunately, we lack the data on newspaper readership in the local markets. Thus, we cannot reliably estimate the degree of media competition. We attempted to use a degree of urbanisation as a proxy (assuming higher degree of media competition in major urban areas than in inner or outer regional areas), but the results were inconclusive due to the level of noise in such a proxy. We leave this important question for further research.

Comment 2
-The description of the 2019 merger seems inconsistent: the intro says "in 2019, Australian entrepreneur Antony Catalano bought Australian Community Media from Nine Entertainment" (who is he and what is his ideology?) and later the paper refers to "Australian Community Media's Acquisition of Nine Entertainment's 257 Regional Newspapers (2019)"

We thank the referee for detecting this inaccuracy. The correct way to describe the deal is "the acquisition of Australia Community Media by Antony Catalano and Alex Waislitz." The paper was edited throughout to ensure consistency.

Comment 3
-I found Figures 4 and 5 very difficult to read. I'd get rid of gridlines, show or at least clearly say when the merger occurred, what each series is (there seem to be three shown but just two in the legend - the series at the bottom of each figure (shaded below in pinkish), which don't go all the way across, seem to not be labeled? And the slants for treatment and control groups are weirdly highly correlated, with many peaks and valleys occurring at the same time...

We thank the referee for their thoughtful comments regarding the clarity of Figures 4 and 5. We have made several changes to improve the readability of these figures.

First, we removed the minor gridlines while keeping the major gridlines to make Figures 4 and 5 more consistent with Figure 1 and to reduce visual clutter. Second, we eliminated the pink shaded area that represented the difference between the treated group and the control group. We recognize that including this area may have overloaded the graph and contributed to confusion, especially since the shaded series at the bottom of the figures were not adequately labelled. Third, we have ensured that the event time (the acquisition) is clearly indicated on the figures. We believe this addresses the concern about clearly showing when the merger occurred. Additionally, we have provided more detailed explanations of each element of the graphs in their legends, explicitly labelling all series depicted.

[10] https://www.waislitzfoundation.org/clontarf-foundation/

We agree with the referee that the plots for the treatment and control groups appear highly correlated, with many peaks and valleys occurring at the same time. This is intentional and a result of the synthetic difference-in-differences (SDID) method we employ, which constructs a synthetic control that closely matches the treated unit's pre-treatment trends. The aim of the SDID method is to ensure that pre-event trends are as parallel as possible, enhancing the validity of the causal inference. We understand the importance of discussing the advantages of the SDID method over a classical difference-in-differences approach, and we have included an additional discussion on this topic on page 11:

> Essentially, synthetic difference-in-differences (Arkhangelky et al, 2021) is a weighted version of the standard two-way fixed effects estimator. It gives more weight to units (like newspapers) that have a similar past to the treated units, and it also highlights time periods that are similar to the treated periods. By focusing on units and periods that are alike, the estimator becomes more robust.

Comment 4

-The regression tables are also unclear. What is the unit of observation - a newspaper-quarter? How many observations are used in each model. These should be stated. If it is newspaper-quarter, I'm not sure if this is right since many newspapers with what should be similar slants over time will be repeated - wouldn't one observation for each newspaper before and after the merger period be better? If newspaper-quarter, should standard errors be clustered by newspaper to account for serial correlation?

We thank the referee for raising these points.

We have edited the descriptions of the tables to clarify that the unit of observation is the newspaper-quarter.

We use full available data for synthetic difference-in-difference (SDID) estimates because the method requires full pre-treatment and post-treatment histories to construct the accurate weights. If we limit the data to just one observation before and after the observation, the method would not work. Intuitively, the method emphasises the newspapers that are more similar in their slant trajectories to the “treated” newspapers. This estimation of similarity in trajectories would be impossible if we limit the data to one pre-treatment period.

The SDID method does not allow for clustering of standard errors. However, we replicated our results with the traditional difference-in-difference method (Table A1) with the clustering of standard errors on the newspaper level, and the results are similar both in magnitude and statistical significance.

(Why jackknife std errors?)

The jackknife procedure for standard errors is used for computational efficiency, It’s recommended by Arkhangelsky et al, (2021) as a computationally efficient alternative to the bootstrap method (the other alternative) as the bootstrap algorithm requires running a computationally costly SDID procedure for every bootstrapped sample. According to Arkhangelsky et al (2021) the jackknife procedure recovers “conservative” standard errors, meaning that the results are unlikely to yield a false positive.

And tests of significant differences across models should be conducted with p-values shown, or the author could interact variables (like High Coalition) with the treatment to test if this leads to a significant diff.

The SDID method does not permit the direct estimation of the interaction variable's effect. However, we calculated the Z-scores for the differences between the coefficients of the respective models using the estimated coefficients and their standard errors. Our analysis revealed that the Z-score was significant in only one instance, and we have accordingly revised the discussion of our results to reflect this finding. We thank the referee for their valuable feedback, which has helped us present our results with greater accuracy.

Comment 5

-I found the introduction to need work, eg it is redundant - it shouldn't state results in the first paragraph and then again on the next page. I also found the reference to the "interquantile range" to be impossible to understand - which quantiles? This range is never referred to again later in the paper.

We have removed the reference to the interquartile range from the first paragraph as suggested. Additionally, we carefully followed the other suggestions, ensuring that the levels of the variables are clearly defined in each table. In the current version, each analysis table reports the 25th percentile, mean, 75th percentile, and standard deviation for the treated group during the pre-event period. These standard deviations are used to estimate the economic magnitudes discussed in the paper. Furthermore, we now report these statistics for both the combined control and treated groups, as well as for the treated group separately in both the pre- and post-event periods. These additional statistics are presented in Table A2, which can be found in the supplementary material.

Comment 6

-The slant verification is quite nice and the lists of top trigrams indeed seem ideologically consistent. The method for measuring slant seems quite similar to Gentzkow and Shapiro (2010) but this is not stated clearly - the parts which are the same as the prior paper should be acknowledged, and innovations or differences should be described clearly too.

We have included the following on page 2:

> *For a sample of more than 200 Australian newspapers and 30 million news articles, we construct a time-varying measure of media bias by comparing the language of the news articles with the speeches of members of Parliament, following the methodology of Gentzkow and Shapiro (2010).*

Comment 7

-Why 150 top trigrams? Perhaps robustness to other numbers should be shown.

In selecting the cut-off, we aimed to balance the breadth of topics covered with the frequency and relevance of trigrams. Intuitively, because we start with the most distinctive trigrams then go further down the trigrams gradually become less and less distinctive between the Coalition and the Labor.

We acknowledge that the choice of 150 trigrams may seem arbitrary, which is why we conducted additional analyses using 50, 100, and 200 trigrams. The results of these analyses are presented in Tables A3, A4, A5 and A6 in the supplementary material. We thank the referee for this valuable comment and have added a clarifying sentence regarding our choice of trigram numbers on page 5 of the paper.

The cut-off of 150 trigrams per period was selected to balance the need for the trigrams to be sufficiently distinctive while also covering a wide variety of topics. Supplementary Material provides robustness checks for our main estimations with 50, 100, and 200 trigrams per party per period.

Reviewer #3:

This paper studies a policy relevant topic in a new and interesting setting. Understanding whether media slant is supply- or demand-driven is relevant for media regulators as market concentration can have opposite effects on slant depending on its source. The highly concentrated newspaper market in Australia, and the period of general decline of print media, present a relevant case study for answering this question.

The paper employs text analysis techniques to quantify slant, and a synthetic difference-in-difference approach to study its dynamics around ownership changes. Overall, the results suggest in the Australian case ownership changes have a significant effect on slant. This adds important nuance to the previous literature, which has focused mostly on the US, and has argued that the role of supply-side factors in determining media slant is relatively small.

Comment 1. a
1. I have two comments on the validation of the text-based measure of slant (which is an important part of the paper).
First, the validation exercise focuses only on the cross-sectional variation in slant across newspapers, rather than its time-varying component. Allowing the slant index to vary over time is the main innovation of this application relative to the original measure developed by Gentzkow-Shapiro 2010. Therefore, I think that the paper should spend more time discussing the time dimension, and should provide evidence (at least anecdotal) that it picks up meaningful over-time variation in the tone of reporting.

We thank the referee for this important suggestion. We have added the following text on page 8:

> It's also important to point out that the dynamics of the slant is consistent with the notable changes in Australia's media landscape. First, we see the gradual movement of *The Australian* to more and more conservative positions between 2010 and 2021. This is consistent with the assessment of former Australian Prime Minister Kevin Rudd, who observed, "If you look at Murdoch's election "news" coverage during the 2007 campaign, it was at best 50-50. Roll the clock to 2010 and 2013, it was more like 80-20 against Labor. Then 90-10 in 2016. And in 2019 it was 100-0."[11]
> Another important feature of the observed dynamics is the left-ward shift of *The Australian* in 2021 as well as the other newspaper around 2019-2020. This is consistent with the increased salience of climate change in Australia following the 2019 election and the bushfires of late 2019 - early 2020, as well as a well-documented editorial shift in the News Corp newspapers regarding the acceptance of the reality of man-made climate change.[12]
>
> A seemingly counterintuitive aspect of the estimated media slants is that *The Australian* in the beginning of the period looks Labor-slanted. It's worth pointing out that we omit the op-eds and only focus on the news articles. This result is consistent with the estimates by Gans and Leigh (2012) who showed that *The Australian* with the op-eds was Coalition-slanted, but *The Australian* without the op-eds was the most left of the centre newspaper among the ten major Australian newspapers they

[11] https://www.theguardian.com/commentisfree/2019/sep/06/democracy-overboard-rupert-murdochs-long-war-on-australian-politics
[12] https://www.nytimes.com/2021/09/06/business/news-corp-climate-change.html

analysed. We see this as an additional validation of our result, since the method of Gans and Leigh (2012), which focused on the mentions of public intellectuals, is significantly different from ours.

Comment 1.b

The slant index is shown to correlate with the share of Labor- vs Coalition-leaning readers. I think that this is reassuring, but different from the definition of "biased reporting" — a newspaper can be read by an audience with a given political orientation but be neutral in its reporting. The ideal validation would be against perceptions of --bias-- in reporting — e.g. a survey that asks people to position the newspaper on the spectrum of Labor-biased / neutral / Coalition-biased.

We thank the referee for this thoughtful remark.

First, we would like to note that our validation measure is not the share of Labor-vs Coalition-leaning readers, but the share of Labor-vs Coalition-leaning readers who get the news from the newspaper because it aligns with their views. We have changed the description of the figures to clarify this.

We would like to provide more context regarding the development of this question. Those questions were co-designed with Roy Morgan (previously Roy Morgan Research) which is one of the oldest and most reputable pollsters in Australia. Our initial version of the validation questions was indeed close to what the referee suggested: a respondent's personal assessments of biassed reporting. The feedback we received from Roy Morgan experts suggested that such questions would hardly be informative for four reasons:

1. Most people would be uninformed of the content of the newspapers they do not read, so they would respond, relying on partisan cues instead of their experience,
2. The word "bias" is negatively charged (and the word "slant" would be unintelligible) so it would likely elicit personal attitude to particular newspapers instead of the assessments of bias as defined in the literature.
3. The popular subjective assessment of the bias can be further contaminated by groupthink and media cues. For example, The Australian is often accused by Labor/Green politicians to be biassed against them, while the conservative politicians often accuse The Age to be biassed in favour of Labor. So, the response would likely pick up this discourse.
4. Finally, partisan respondents tend to believe that the media are biassed against their side. This would add additional noise to the measure.

The suggested ways to mitigate this issue was to limit respondents for a particular newspaper to the readers of that newspaper and to ask a neutrally formulated question about the perceived congruence of the content of the newspaper with the respondent's political beliefs. This is exactly what we have done and reported in the paper.

Having said that, the perceived bias can be an important way to validate our measure if it's assessed by media experts. Our results are consistent with the bias assessment by Media Bias/Fact Check, an independent website founded in 2015 by Dave van Zandt. For example, MBFC reports The Australian as Center-Right, and Sydney Morning Herald and the Age as Center-Left. Unfortunately, the sample of Australian newspapers in the MBFC data are too small to run a meaningful correlation analysis with our scores.

Comment 2
For transparency, I would recommend reporting the mix of newspapers picked up in the synthetic control method for each treated newspaper along with their respective weights.
It was also not entirely clear why the synthetic control was preferred over a more standard difference-in-difference method using all untreated newspapers as control units. If the concern are common trends in slant (as suggested by Figure 1), why not simply difference them out with time effects?

We thank the referee for the suggestion and have reported all weights for the synthetic control group in the supplementary materials (Tables A7, A8, A9 and A10). For the reader's convenience, the newspapers have been ordered by weight in decreasing order. The distribution of weights varies significantly, providing a more precise and accurate estimate of the effect.

We also repeated our analysis using a simple difference-in-difference (DiD) approach and reported the estimates in Table A1. We find that the results of DiD are similar, in terms of sign and significance, to those of SDID. However, we believe that the SDID approach provides a more reliable estimate in our setting, as it better ensures that the trends are more parallel.

Comment 3
It would also be useful to report the baseline levels of the slant in each table and give a better sense of the magnitude of the estimated effects and how they compare to the literature.

We have updated the format of all tables and included the reporting of mean, standard deviation, and the 25th and 75th percentiles. We determined that it is best to report these summary statistics for the pre-event sample of the treatment group so that the reader can assess the effect's magnitude relative to the earlier period for the group that would later be affected. Additionally, we provide similar summary statistics for the full sample (treatment and control groups during the full SDID period) and the complete treatment sample (only the treatment group before and after the acquisition) in the supplementary material (Table A2).

We have also added the comparison with Garz and Rickardsson (2023) which is the closest paper in the literature to ours on page 13:

> The estimates are close to what Garz and Rickardsson (2023) provide as the correlation between the estimated slant and the newspaper ideology (23.2% of observed slant).

Comment 4
I think that the paper could provide more context on the political orientation of the owners of interest. This would be helpful for readers who are not familiar with the Australian market.

We thank the referee for this concern. We have added this discussion on page 10:

> News Corp is the largest owner of print media in Australia. Its proprietor and executive chairman (2013-2023) Rupert Murdoch is described as "staunch conservative"[13] and the outlets associated with

[13] https://www.politico.eu/article/rupert-murdoch-dominated-uk-politics-last-his-kind-fox-news-coproration-media/

him are described to exhibit a leaning towards a conservative side of the political spectrum (Martin and Yurukoglu, 2017).

Fairfax Media was one of the oldest media companies in Australia. In 2012, after the Fairfax family sold its stake,[14] Gina Rinehart, Australian mining magnate known for her support for conservative politicians[15] and opposition to carbon tax,[16] became its largest shareholder. She sold her Fairfax stake in 2015.[17] Fairfax Media itself was not named as providing funds to political parties since 2012.

Nine Entertainment is a publicly traded company with a diverse set of shareholders. According to Australian Electoral Commission, in 2012-2019, Nine Entertainment was named as providing funds to Australian Labor Party (more than 50,000 AUD over 7 years).[18] It should be also noted Fairfax journalists had adopted a charter asserting their independence.[19] It was also reported that the charter remained unsigned by Nine executives.[20]

Australian Community Media is a media company responsible for a set of regional newspapers. It has been a part of Fairfax Media since 2007, then a part of Nine Entertainment (since the Nine/Fairfax merger in 2018). In 2018, it was bought and Antony Catalano and Alex Waislitz.[21]

The new owners of Australian Community Media, Antony Catalano and Alex Waislitz, refrain from publicly expressing their political views, emphasising instead the business aspects in their appearances and interviews. Notably, Alex Waislitz's philanthropic foundation has partnered with the Clontarf Foundation, an organisation dedicated to enhancing opportunities for Indigenous children.[22] It is also important to acknowledge that phrases surrounding Indigenous issues are associated with the speeches of Labor Party politicians. Neither Catalano nor Waislitz are shown on the returns of the Australian Electoral Commission as donors around the time of the acquisition.

---

14 https://www.smh.com.au/business/fairfax-family-sells-out-of-company-20111110-1n96f.html
15 https://www.ginarinehart.com.au/gina-rinehart-at-the-conservative-political-action-conference-in-maryland-on-saturday/
16 https://www.theguardian.com/environment/blog/2011/may/31/mining-media-australia-carbon-tax
17 https://www.smh.com.au/business/companies/gina-rinehart-sells-out-of-fairfax-media-20150206-138b5i.html
18 https://transparency.aec.gov.au/ClientEntity/EntityDetail?clientIdentifier=21242&eventId=&clientType=organisationdonor
19 https://www.smh.com.au/national/fairfax-media-charter-of-editorial-independence-20120619-20l4t.html
20 https://theklaxon.com.au/home-nine-fairfax-independence-charter-a-sham-ecscp/
21 https://www.abc.net.au/news/2019-04-30/nine-sells-fairfax-community-newspapers-to-the-cat/11058066
22 https://www.waislitzfoundation.org/clontarf-foundation/

Table A1. Robustness: Regular Difference-in-Differnece Analysis

This table estimates a standard difference-in-differences specification as a robustness check to the synthetic difference-in-differences analysis presented in the main body of the paper. Specifically, we follow the same steps to construct treatment and control groups, then estimate the following regression:

$$Slant_{n,t} = Treated_{n,t} \times Post_{n,t} + \kappa_n + \zeta_t + \varepsilon_{n,t}$$

In this specification, κ_n represents newspaper fixed effects, and ζ_t represents time fixed effects (FE), both of which absorb the individual treatment and post-period coefficients. Standard errors are clustered at the newspaper level to account for within-newspaper correlation. Standard errors are reported in parentheses. *** indicates p-value < 0.01, ** indicates p-value < 0.05, and * indicates p-value < 0.1.

(a) Difference-in-Differences Analysis Based on Table 2

	All States	NSW State	QLD State	High Coalition
Treated × Post	-0.023	0.015	-0.014	-0.003
	(0.066)	(0.132)	(0.146)	(0.087)
Num.Obs.	2,898	1,638	234	1,710
R2	0.321	0.273	0.507	0.247
FE: newspaper	X	X	X	X
FE: quarter	X	X	X	X

(b) Difference-in-Differences Analysis Based on Table 3

	All States	NSW State	QLD State	SA State	High Coalition	Low Coalition
Treated × Post	-0.123***	-0.164**	0.128	0.166	-0.189***	-0.065
	(0.042)	(0.067)	(0.121)	(0.119)	(0.064)	(0.057)
Num.Obs.	4,752	2,034	630	468	2,106	2,340
R2	0.313	0.281	0.277	0.341	0.347	0.263
FE: newspaper	X	X	X	X	X	X
FE: quarter	X	X	X	X	X	X

(c) Difference-in-Differences Analysis Based on Table 4

	All States	NSW State	VIC State	QLD State	High Coalition	Low Coalition
Treated × Post	0.206***	0.254***	0.348***	0.093	0.229***	0.200***
	(0.039)	(0.057)	(0.076)	(0.064)	(0.058)	(0.055)
Num.Obs.	4,063	1,785	833	578	1,836	2,074
R2	0.344	0.330	0.398	0.372	0.356	0.312
FE: newspaper	X	X	X	X	X	X
FE: quarter	X	X	X	X	X	X

(d) Difference-in-Differences Analysis Based on Table 5

	(1) All States	(2) NSW State	(3) VIC State	(4) QLD State	(5) High Coalition	(6) Low Coalition
Treated × Post	0.192***	0.245***	0.310**	-0.037	0.237***	0.167***
	(0.039)	(0.058)	(0.118)	(0.064)	(0.061)	(0.054)
Num.Obs.	3,978	1,782	720	630	1,782	2,034
R2	0.319	0.290	0.402	0.332	0.333	0.295
FE: newspaper	X	X	X	X	X	X
FE: time	X	X	X	X	X	X

Table A2. Additional Summary Statistics

This table provides additional summary statistics for Tables 2, 3, 4, and 5. Specifically, the first four rows of each panel present the 25th percentile, mean, 75th percentile, and standard deviation for all observations in the treatment and pooled synthetic control groups across all (pre- and post-) periods. The last four rows present statistics for the treatment group only, covering both the pre- and post-event periods.

(a) Additional Summary Statistics for Table 2

	All States	NSW	QLD	High Coal
**Full Sample**				
Q1	-0.345	-0.382	-0.319	-0.444
Mean	-0.059	-0.049	-0.036	-0.126
Q3	0.254	0.289	0.269	0.196
SD	0.550	0.565	0.477	0.551
**Treated Subsample**				
Q1	-0.018	-0.112	-0.052	-0.083
Mean	0.132	0.072	0.178	0.132
Q3	0.281	0.274	0.382	0.320
SD	0.332	0.336	0.378	0.376

(b) Additional Summary Statistics for Table 3

	All States	NSW	QLD	VIC	SA	High Coal	Low Coal
**Full Sample**							
Q1	-0.333	-0.333	-0.503	0	-0.577	-0.167	-0.448
Mean	-0.016	-0.029	-0.171	0.251	-0.246	0.094	-0.108
Q3	0.333	0.333	0.127	0.625	0.037	0.409	0.222
SD	0.564	0.564	0.493	0.583	0.536	0.542	0.573
**Treated Subsample**							
Q1	-0.222	-0.222	-0.467	0	-0.330	-0.110	-0.372
Mean	0.060	0.055	-0.136	0.277	-0.084	0.121	-0.013
Q3	0.337	0.370	0.217	0.667	0.111	0.389	0.333
SD	0.554	0.526	0.497	0.607	0.416	0.533	0.583

(c) Additional Summary Statistics for Table 4

	All States	NSW	VIC	QLD	Low Coal	High Coal
**Full Sample**						
Q1	-0.222	-0.295	0	-0.328	-0.030	-0.384
Mean	0.028	0.036	0.193	-0.020	0.121	-0.054
Q3	0.333	0.374	0.447	0.233	0.400	0.259
SD	0.521	0.559	0.438	0.490	0.490	0.540
**Treated Subsample**						
Q1	-0.389	-0.359	-0.027	-0.672	-0.169	-0.500
Mean	-0.044	0.005	0.125	-0.38	0.099	-0.134
Q3	0.333	0.389	0.453	-0.105	0.455	0.208
SD	0.567	0.595	0.45	0.438	0.545	0.563

(d) Additional Summary Statistics for Table 5

	All States	NSW	VIC	QLD	Low Coal	High Coal
**Full Sample**						
Q1	-0.249	-0.278	0	-0.322	-0.066	-0.388
Mean	-0.006	0.005	0.128	-0.037	0.078	-0.080
Q3	0.280	0.333	0.333	0.210	0.333	0.222
SD	0.509	0.522	0.454	0.476	0.482	0.523
**Treated Subsample**						
Q1	-0.436	-0.380	-0.305	-0.617	-0.289	-0.500
Mean	-0.082	-0.025	0.069	-0.330	0.039	-0.154
Q3	0.307	0.333	0.501	-0.004	0.408	0.207
SD	0.566	0.555	0.537	0.443	0.574	0.550

Table A3. Robustness Check: Alternative Trigram Selection for Political Slant (Based on Table 2)

This table re-estimates the analysis from Table 2, using a political slant measure based on a different number of trigrams, rather than the 150 trigrams used in the main analysis.

(a) Variation with 50 Trigrams

Sample	All States	NSW	QLD	High Co
Coefficient	0.007	-0.041	-0.003	0.012
Std. Error	0.035	0.103	0.08	0.056
N. Obs	2700	1638	234	1710

(b) Variation with 100 Trigrams

Sample	All States	NSW	QLD	High Co
Coefficient	0.028	0.056	0.146*	0.071
Std. Error	0.042	0.133	0.078	0.059
N. Obs	2700	1638	234	1710

(c) Variation with 200 Trigrams

Sample	All States	NSW	QLD	High Co
Coefficient	-0.006	-0.119	0.044	0.02
Std. Error	0.044	0.15	0.071	0.067
N. Obs	2700	1620	252	1710

Table A4. Robustness Check: Alternative Trigram Selection for Political Slant (Based on Table 3)

This table re-estimates the analysis from Table 3, using a political slant measure based on a different number of trigrams, rather than the 150 trigrams used in the main analysis.

(a) Variation with 50 Trigrams

Sample	All	NSW	QLD	VIC	SA	Low Co	High Co
Coefficient	-0.064**	-0.037	0.07	-0.104***	0.259***	-0.108**	-0.026
Std. Error	0.032	0.047	0.077	0.038	0.079	0.042	0.049
N. Obs	4482	2016	630	936	468	2088	2340

(b) Variation with 100 Trigrams

Sample	All	NSW	QLD	VIC	SA	Low Co	High Co
Coefficient	-0.067*	-0.091	0.264***	-0.192***	0.202***	-0.18***	-0.012
Std. Error	0.035	0.056	0.067	0.044	0.077	0.049	0.047
N. Obs	4482	2016	630	936	468	2088	2340

(c) Variation with 200 Trigrams

Sample	All	NSW	QLD	VIC	SA	Low Co	High Co
Coefficient	-0.046	-0.042	0.161***	-0.343***	0.257***	-0.206***	0.093*
Std. Error	0.036	0.048	0.048	0.052	0.082	0.047	0.053
N. Obs	4518	2052	648	936	450	2088	2376

Table A5. Robustness Check: Alternative Trigram Selection for Political Slant (Based on Table 4)

This table re-estimates the analysis from Table 4, using a political slant measure based on a different number of trigrams, rather than the 150 trigrams used in the main analysis.

(a) Variation with 50 Trigrams

Sample	All	NSW	VIC	QLD	Low Co	High Co
Coefficient	0.074**	0.065	-0.006	0	0.004	0.108***
Std. Error	0.032	0.042	0.058	0.236	0.048	0.04
N. Obs	3876	1751	816	578	1819	2006

(b) Variation with 100 Trigrams

Sample	All	NSW	VIC	QLD	Low Co	High Co
Coefficient	0.116***	0.151***	0.034	-0.021	0.081*	0.11***
Std. Error	0.029	0.049	0.037	0.166	0.047	0.038
N. Obs	3927	1785	816	578	1819	2057

(c) Variation with 200 Trigrams

Sample	All	NSW	VIC	QLD	Low Co	High Co
Coefficient	0.131***	0.139***	0.142**	-0.016	0.116***	0.156***
Std. Error	0.028	0.037	0.068	0.128	0.037	0.042
N. Obs	4029	1853	833	578	1870	2108

Table A6. Robustness Check: Alternative Trigram Selection for Political Slant (Based on Table 5)

This table re-estimates the analysis from Table 5, using a political slant measure based on a different number of trigrams, rather than the 150 trigrams used in the main analysis.

(a) Variation with 50 Trigrams

Sample	All	NSW	VIC	QLD	Low Co	High Co
Coefficient	0.057*	-0.095***	0.083	0.028	0.003	0.057
Std. Error	0.034	0.031	0.091	0.121	0.053	0.042
N. Obs	3621	1666	680	595	1666	1904

(b) Variation with 100 Trigrams

Sample	All	NSW	VIC	QLD	Low Co	High Co
Coefficient	0.07**	0.053	0.006	-0.103	0.022	0.082**
Std. Error	0.028	0.044	0.138	0.109	0.041	0.041
N. Obs	3638	1666	697	595	1666	1921

(c) Variation with 200 Trigrams

Sample	All	NSW	VIC	QLD	Low Co	High Co
Coefficient	0.076***	0.096**	0.171	-0.018	0.116**	0.053
Std. Error	0.028	0.041	0.15	0.068	0.048	0.039
N. Obs	3672	1700	697	595	1666	1955

Table A7. Weights of the Synthetic Control Group (Based on Table 2)

This table reports the weights assigned to newspapers in the synthetic control group used for the analysis in Table 2.

Newspaper	Weight	Newspaper	Weight
Merimbula News Weekly	0.0101	Blacktown City Sun	0.0069
The Northern Argus	0.0098	The Northern Daily Leader	0.0068
Port Lincoln Times	0.0097	The North Queensland Register	0.0068
Maribyrnong & Hobsons Bay Weekly	0.0093	The Narromine News	0.0067
Singleton Argus	0.0093	Southern Cross	0.0067
Merredin – Wheatbelt Mercury	0.0092	Narooma News	0.0067
Donnybrook – Bridgetown Mail	0.0090	The Rural	0.0067
The Camden Haven Courier	0.0090	The Standard	0.0067
Wauchope Gazette	0.0090	Wingham Chronicle	0.0066
Hills News	0.0088	The Islander	0.0066
Moree Champion	0.0087	The Flinders News	0.0066
Bega District News	0.0087	Central Midlands and Coastal Advocate	0.0066
Maitland Mercury	0.0087	Eastern Riverina Chronicle	0.0066
The Manning River Times	0.0085	Wyndham Weekly	0.0066
Augusta – Margaret River Mail	0.0084	The Boorowa News	0.0066
The Irrigator	0.0084	The Transcontinental	0.0065
Redland City Bulletin	0.0084	Southern Weekly	0.0065
Magnet	0.0083	Liverpool City Champion	0.0065
Stock and Land	0.0083	Stock Journal	0.0065
Milton Ulladulla Times	0.0082	The Age	0.0065
Bunbury Mail	0.0082	Kiama Independent	0.0064
Northern Weekly	0.0082	Illawarra Mercury	0.0064
The Inverell Times	0.0080	The Murray Valley Standard	0.0064
Melton & Moorabool Weekly	0.0080	Cootamundra Herald	0.0064
The Naracoorte Herald	0.0080	The Grenfell Record and Bland Advertiser	0.0064
Port Macquarie News	0.0080	Western Advocate	0.0064
Sunbury & Macedon Ranges Weekly	0.0079	Daily Liberal and Macquarie Advocate	0.0064
Glen Innes Examiner	0.0079	Eyre's Peninsula Tribune	0.0063
The Recorder	0.0079	The Land	0.0063
Hawkesbury Gazette	0.0078	The Canberra Times	0.0062
Crookwell Gazette	0.0077	Coastal Leader	0.0062
The Young Witness	0.0077	Town & Country Magazine	0.0062
The Dungog Chronicle	0.0076	Penrith City Gazette	0.0062
The Tenterfield Star	0.0075	Mandurah Mail	0.0062
Goulburn Post	0.0075	The Collie Mail	0.0062
The Examiner	0.0075	Sunday Age	0.0061
St. George and Sutherland Shire Leader	0.0075	Herald	0.0061
The Avon Valley Advocate	0.0075	Western Magazine	0.0061
Northern News	0.0075	Braidwood Times	0.0061
Blayney Chronicle	0.0075	Lithgow Mercury	0.0061
Queensland Country Life	0.0075	The Bellingen Shire Courier – Sun	0.0060
The Sun (Parramatta, Holroyd)	0.0074	The Border Chronicle	0.0060
Southern Highland News	0.0074	The Advertiser	0.0060
The North West Star	0.0074	Nyngan Observer	0.0060
The Queanbeyan Age	0.0074	The Cowra Guardian	0.0059
Port Stephens Examiner	0.0074	Roxby Downs Sun	0.0059
Whyalla News	0.0074	The Bendigo Advertiser	0.0058
Brimbank & North West Weekly	0.0074	Bombala Times	0.0058
The Esperance Express	0.0073	The Times	0.0057
Advocate	0.0073	Blue Mountains Gazette	0.0057
The Harden – Murrumburrah Express	0.0073	The Wimmera Mail – Times	0.0057
South West Advertiser	0.0073	Great Lakes Advocate	0.0056
Advocate	0.0072	Wellington Times	0.0056
The Advertiser	0.0072	Oberon Review	0.0055
The Courier	0.0072	The Goondiwindi Argus	0.0055
The Scone Advocate	0.0072	Yass Tribune	0.0054
The Daily Advertiser	0.0071	The Walcha News	0.0053
Katherine Times	0.0071	Bay Post	0.0053
Bayside Bulletin	0.0071	Ararat Advertiser and Stawell Times	0.0053
Central Western Daily	0.0071	Area News	0.0053
The Armidale Express	0.0071	The Border Mail	0.0052
Fairfield City Champion	0.0071	Guyra Argus	0.0050
Busselton – Dunsborough Mail	0.0071	The Star	0.0049
Sydney Morning Herald	0.0070	Cooma Monaro Express	0.0048
Forbes Advocate	0.0070	Macleay Argus	0.0048
South Coast Register	0.0070	Canowindra News	0.0047
St. Marys Star	0.0070	Parkes Champion Post	0.0047
The Gloucester Advocate	0.0070	Wollondilly Advertiser	0.0045
The West Coast Sentinel	0.0070	The Ridge News	0.0044
Farm Weekly	0.0069	Mudgee Guardian and Gulgong Advertiser	0.0042
Sun Herald	0.0069	Campbelltown – Macarthur Advertiser	0.0042
The Muswellbrook Chronicle	0.0069	Camden Advertiser	0.0042
Guardian News	0.0069		

Table A8. Weights of the Synthetic Control Group (Based on Table 3)

This table reports the weights assigned to newspapers in the synthetic control group used for the analysis in Table 3.

Newspaper	Weight	Newspaper	Weight
Goulburn Post	0.0102	The Walcha News	0.0069
Northern News	0.0099	Farm Weekly	0.0068
Bombala Times	0.0098	Melton & Moorabool Weekly	0.0068
Sunday Age	0.0096	The Bellingen Shire Courier - Sun	0.0068
The Islander	0.0096	St. Marys Star	0.0068
The Queanbeyan Age	0.0096	The Boorowa News	0.0067
Merimbula News Weekly	0.0094	The North West Star	0.0067
The Irrigator	0.0094	Blue Mountains Gazette	0.0067
The Northern Argus	0.0093	Cootamundra Herald	0.0067
The Muswellbrook Chronicle	0.0093	Guardian News	0.0066
Parkes Champion Post	0.0092	The Northern Daily Leader	0.0066
The Armidale Express	0.0092	The Canberra Times	0.0066
Sun Herald	0.0092	The Grenfell Record and Bland Advertiser	0.0066
Northern Weekly	0.0090	Town & Country Magazine	0.0066
Central Western Daily	0.0090	Bayside Bulletin	0.0066
Cooma Monaro Express	0.0088	Yass Tribune	0.0066
The Camden Haven Courier	0.0088	Oberon Review	0.0066
Great Lakes Advocate	0.0088	Advocate	0.0066
Fairfield City Champion	0.0088	The Rural	0.0065
South Coast Register	0.0087	Lithgow Mercury	0.0065
The Manning River Times	0.0086	Wollondilly Advertiser	0.0065
Daily Liberal and Macquarie Advocate	0.0085	Sydney Morning Herald	0.0065
Hills News	0.0083	The Border Chronicle	0.0064
Port Lincoln Times	0.0082	Central Midlands and Coastal Advocate	0.0064
The Naracoorte Herald	0.0081	The Tenterfield Star	0.0064
The Sun (Parramatta, Holroyd)	0.0081	The Narromine News	0.0064
The Dungog Chronicle	0.0080	The Gloucester Advocate	0.0064
The Murray Valley Standard	0.0080	Guyra Argus	0.0063
Redland City Bulletin	0.0079	Maitland Mercury	0.0062
Wingham Chronicle	0.0079	The Standard	0.0062
Blayney Chronicle	0.0079	Eyre's Peninsula Tribune	0.0062
Eastern Riverina Chronicle	0.0079	Campbelltown - Macarthur Advertiser	0.0062
The Daily Advertiser	0.0079	Brimbank & North West Weekly	0.0062
St. George and Sutherland Shire Leader	0.0078	Canowindra News	0.0061
Busselton - Dunsborough Mail	0.0078	The Goondiwindi Argus	0.0061
Forbes Advocate	0.0077	The Esperance Express	0.0061
The Collie Mail	0.0077	Area News	0.0059
Stock Journal	0.0077	Wyndham Weekly	0.0059
Blacktown City Sun	0.0077	The Star	0.0059
Queensland Country Life	0.0077	Herald	0.0058
Port Stephens Examiner	0.0076	The Bendigo Advertiser	0.0058
Donnybrook - Bridgetown Mail	0.0076	Bunbury Mail	0.0057
Southern Cross	0.0076	Mudgee Guardian and Gulgong Advertiser	0.0057
Katherine Times	0.0076	Mandurah Mail	0.0057
Southern Weekly	0.0076	The Transcontinental	0.0057
The Harden - Murrumburrah Express	0.0075	Macleay Argus	0.0057
Glen Innes Examiner	0.0075	Nyngan Observer	0.0057
Merredin - Wheatbelt Mercury	0.0075	Magnet	0.0056
Crookwell Gazette	0.0074	Whyalla News	0.0056
The Flinders News	0.0074	Milton Ulladulla Times	0.0056
The Recorder	0.0074	Narooma News	0.0055
The Cowra Guardian	0.0073	The Avon Valley Advocate	0.0054
The North Queensland Register	0.0073	Ararat Advertiser and Stawell Times	0.0054
Singleton Argus	0.0072	Bega District News	0.0052
Herald	0.0072	The Scone Advocate	0.0052
The Inverell Times	0.0072	Maribyrnong & Hobsons Bay Weekly	0.0051
The Times	0.0071	The Border Mail	0.0051
Advocate	0.0071	Southern Highland News	0.0050
The Advertiser	0.0071	Western Advocate	0.0050
The Courier	0.0071	The Ridge News	0.0050
The Advertiser	0.0071	Wauchope Gazette	0.0047
Illawarra Mercury	0.0071	The Wimmera Mail - Times	0.0047
The Age	0.0070	Camden Advertiser	0.0045
South West Advertiser	0.0070	Port Macquarie News	0.0043
Liverpool City Champion	0.0070	Kiama Independent	0.0040
Penrith City Gazette	0.0070	Wellington Times	0.0039
The Land	0.0069	Roxby Downs Sun	0.0038
Augusta - Margaret River Mail	0.0069	Moree Champion	0.0037
Stock and Land	0.0069	The Young Witness	0.0034
Hawkesbury Gazette	0.0069	The West Coast Sentinel	0.0034
Western Magazine	0.0069	Sunbury & Macedon Ranges Weekly	0.0034
Braidwood Times	0.0069	Coastal Leader	0.0034
The Examiner	0.0069	Bay Post	0.0034

Table A9. Weights of the Synthetic Control Group (Based on Table 4)

This table reports the weights assigned to newspapers in the synthetic control group used for the analysis in Table 4.

**Newspaper**	**Weight**	**Newspaper**	**Weight**
Fairfield Advance	0.0114	Darwin Palmerston Sun	0.0082
The Albert and Logan News	0.0111	Echo	0.0082
Central Coast Express Advocate	0.0108	Southern Courier	0.0081
Brisbane News	0.0101	Maroondah Leader	0.0081
The Manly Daily	0.0101	Mornington Peninsula Leader	0.0081
Penrith Press	0.0099	The Cairns Post	0.0081
Rouse Hill Times	0.0099	Mt. Druitt - St. Mary's Standard	0.0081
Leader Messenger	0.0098	Greater Dandenong Leader	0.0081
Southern Weekly	0.0097	The Southern Star	0.0081
Bribie Weekly	0.0097	Midland Kalamunda Reporter	0.0080
Parramatta Advertiser	0.0096	Free Press Leader	0.0080
Tablelands Advertiser	0.0095	Moonee Valley Leader	0.0080
Blacktown Advocate	0.0095	The Herbert River Express	0.0080
City Messenger	0.0094	Wyndham Leader	0.0080
Inner - West Weekly	0.0093	Wynnum Herald	0.0080
North Shore Times	0.0093	Whittlesea Leader	0.0079
The Hills Shire Times	0.0093	Eastern Courier Messenger	0.0079
The Weekend Australian Magazine	0.0092	Townsville Sun	0.0079
Tasmanian Country	0.0092	Sunday Tasmanian	0.0079
Moreland Leader	0.0092	City North Messenger	0.0079
The Atherton Tablelander	0.0091	Melton Leader	0.0079
Mosman Daily	0.0090	Cooma Monaro Express	0.0079
The Leader	0.0090	The Northern Territory News	0.0079
Townsville Bulletin	0.0090	Geelong Advertiser	0.0079
Guardian Messenger	0.0090	Penrith City Gazette	0.0078
The Courier - Mail	0.0088	Progress Leader	0.0077
Wish	0.0088	Caboolture Shire Herald	0.0077
The Australian	0.0088	Weekly Times Messenger	0.0077
Weekend Australian	0.0088	The Daily Telegraph	0.0077
Sportsman	0.0087	Comment News	0.0076
City North News	0.0087	The Express	0.0076
The Weekly Times	0.0087	East Torrens Messenger	0.0076
The Mercury	0.0087	Portside Messenger	0.0076
Blacktown City Sun	0.0087	Wentworth Courier	0.0075
Northern District Times	0.0087	Innisfail Advocate	0.0075
Hills News	0.0086	Gold Coast Sun	0.0075
The Gold Coast Bulletin	0.0086	Melbourne Yarra Leader	0.0075
North Coast Times	0.0086	Moorabbin Glen Eira Kingston Leader	0.0075
North - West News	0.0086	Hobsons Bay Leader	0.0075
Bayside Leader	0.0086	Bowen Independent	0.0075
Macarthur Chronicle	0.0085	Heidelberg Leader	0.0074
Central	0.0085	Mordialloc - Chelsea Leader	0.0074
Pine Rivers Press	0.0085	Hills Gazette	0.0073
Hornsby and Upper North Shore Advocate	0.0085	Monash Leader	0.0073
The Redcliffe & Bayside Herald	0.0085	Northcote Leader	0.0073
Geelong News	0.0085	Whitehorse Leader	0.0072
Port Douglas & Mossman Gazette	0.0085	Northern Miner	0.0072
Southern Times Messenger	0.0085	City South News	0.0072
Herald Sun	0.0085	Maribyrnong Leader	0.0072
Sunday Telegraph	0.0085	Brimbank Leader	0.0072
The Sun (Parramatta, Holroyd)	0.0084	Eastern Riverina Chronicle	0.0070
Inner West Courier	0.0084	Knox Leader	0.0070
Hume Leader	0.0084	Stonnington Leader	0.0067
Sunbury Macedon Ranges Leader	0.0084	South - East Advertiser	0.0067
Berwick / Pakenham Cardinia Leader	0.0083	Westside News	0.0066
Manningham Leader	0.0083	The Cairns Sun	0.0066
Frankston Standard Leader / Hastings Leader	0.0083	Preston Leader	0.0066
South West News	0.0083	Lilydale Yarra Valley Leader	0.0064
News Mail Bundaberg	0.0083	The Burdekin Advocate	0.0061
St. Marys Star	0.0083	Cranbourne Leader	0.0059
The Centralian Advocate	0.0082		

Table A10. Weights of the Synthetic Control Group (Based on Table 5)

This table reports the weights assigned to newspapers in the synthetic control group used for the analysis in Table 5.

Newspaper	Weight	Newspaper	Weight
The Atherton Tablelander	0.0143	Portside Messenger	0.0087
Echo	0.0128	Central Coast Express Advocate	0.0086
North Coast Times	0.0127	Southern Courier	0.0086
Southern Weekly	0.0126	City North Messenger	0.0085
Pine Rivers Press	0.0124	St. Marys Star	0.0083
Tablelands Advertiser	0.0123	East Torrens Messenger	0.0083
The Albert and Logan News	0.0118	The Burdekin Advocate	0.0082
Comment News	0.0118	Mosman Daily	0.0082
The Cairns Post	0.0116	The Northern Territory News	0.0081
The Weekend Australian Magazine	0.0115	Hills Gazette	0.0081
The Gold Coast Bulletin	0.0115	South West News	0.0081
Hume Leader	0.0114	The Express	0.0081
Caboolture Shire Herald	0.0114	Inner West Courier	0.0081
The Weekly Times	0.0114	Gold Coast Sun	0.0080
Weekend Australian	0.0113	Sunday Telegraph	0.0080
Bribie Weekly	0.0112	Moreland Leader	0.0079
News Mail Bundaberg	0.0111	Wentworth Courier	0.0078
Farm Weekly	0.0110	Weekly Times Messenger	0.0077
Sunday Tasmanian	0.0109	North - West News	0.0077
Hills News	0.0107	The Manly Daily	0.0076
Tasmanian Country	0.0106	Progress Leader	0.0075
Brisbane News	0.0105	Moonee Valley Leader	0.0075
Northern Miner	0.0104	Sunbury Macedon Ranges Leader	0.0074
The Daily Telegraph	0.0104	Greater Dandenong Leader	0.0074
Townsville Bulletin	0.0104	Southern Times Messenger	0.0073
Berwick / Pakenham Cardinia Leader	0.0102	Inner - West Weekly	0.0073
The Australian	0.0101	Eastern Courier Messenger	0.0073
Bowen Independent	0.0101	The Cairns Sun	0.0072
Sportsman	0.0100	The Herbert River Express	0.0071
Leader Messenger	0.0100	Rouse Hill Times	0.0067
Herald Sun	0.0100	The Leader	0.0066
Geelong News	0.0099	Fairfield Advance	0.0066
Blacktown Advocate	0.0099	South - East Advertiser	0.0064
The Redcliffe & Bayside Herald	0.0098	Moorabbin Glen Eira Kingston Leader	0.0061
Guardian Messenger	0.0098	Darwin Palmerston Sun	0.0059
The Mercury	0.0097	Knox Leader	0.0059
The Sun (Parramatta, Holroyd)	0.0097	Westside News	0.0058
The Centralian Advocate	0.0095	Bayside Leader	0.0058
Mornington Peninsula Leader	0.0094	The Hills Shire Times	0.0057
Melbourne Yarra Leader	0.0094	Macarthur Chronicle	0.0057
The Southern Star	0.0094	North Shore Times	0.0055
Port Douglas & Mossman Gazette	0.0094	Penrith Press	0.0052
Hobsons Bay Leader	0.0093	Whittlesea Leader	0.0052
Frankston Standard Leader / Hastings Leader	0.0092	Central	0.0052
Wyndham Leader	0.0092	Whitehorse Leader	0.0050
Brimbank Leader	0.0092	Heidelberg Leader	0.0050
Geelong Advertiser	0.0092	City North News	0.0049
Townsville Sun	0.0091	Maribyrnong Leader	0.0047
Blacktown City Sun	0.0091	Monash Leader	0.0045
Melton Leader	0.0090	Preston Leader	0.0044
The Courier - Mail	0.0090	Northcote Leader	0.0042
Penrith City Gazette	0.0090	Mordialloc - Chelsea Leader	0.0041
Wynnum Herald	0.0090	Stonnington Leader	0.0041
Mt. Druitt - St. Mary's Standard	0.0090	Northern District Times	0.0041
Innisfail Advocate	0.0090	Cranbourne Leader	0.0037
Midland Kalamunda Reporter	0.0089	Lilydale Yarra Valley Leader	0.0036
Eastern Riverina Chronicle	0.0089	Maroondah Leader	0.0035
Wish	0.0089	Manningham Leader	0.0035
Parramatta Advertiser	0.0088	Hornsby and Upper North Shore Advocate	0.0035
City Messenger	0.0087	City South News	0.0035
