# Supplementary material for: Media ownership and ideological slant: Evidence from Australian newspaper mergers
Source: PLoS One. 2024 Dec 31;19(12):e0315137. doi: 10.1371/journal.pone.0315137 (PMC11687783; doi:10.1371/journal.pone.0315137)
Supplement: Peer review history

# Peer review: Decision Letter 0

**DOI:** 10.1371/journal.pone.0315137.r001

**Authors:** Simon Porcher

26 Aug 2024

PONE-D-24-27523Media Ownership and Ideological Slant: Evidence from Australian Newspaper MergersPLOS ONE

Dear Dr. Volkova,

Thank you for submitting your manuscript to PLOS ONE. After careful consideration, we feel that it has merit but does not fully meet PLOS ONE’s publication criteria as it currently stands. Therefore, we invite you to submit a revised version of the manuscript that addresses the points raised during the review process.

 The reviewers and I much enjoyed your paper which make a solid contribution. However, reviewers have pointed some minor changes to be added to the current version of the manuscript. These changes will improve the narratives. Reviewer 3 has also some comments on the empircal part of the paper to improve the transparency of your research. Adding some vignettes or anecdotes could be useful to illustrate the robustness of your index and dataset. 

Please submit your revised manuscript by Oct 05 2024 11:59PM. If you will need more time than this to complete your revisions, please reply to this message or contact the journal office at plosone@plos.org. When you're ready to submit your revision, log on to https://www.editorialmanager.com/pone/ and select the 'Submissions Needing Revision' folder to locate your manuscript file.

Please include the following items when submitting your revised manuscript:

- A rebuttal letter that responds to each point raised by the academic editor and reviewer(s). You should upload this letter as a separate file labeled 'Response to Reviewers'.
- A marked-up copy of your manuscript that highlights changes made to the original version. You should upload this as a separate file labeled 'Revised Manuscript with Track Changes'.
- An unmarked version of your revised paper without tracked changes. You should upload this as a separate file labeled 'Manuscript'.

If you would like to make changes to your financial disclosure, please include your updated statement in your cover letter. Guidelines for resubmitting your figure files are available below the reviewer comments at the end of this letter.

If applicable, we recommend that you deposit your laboratory protocols in protocols.io to enhance the reproducibility of your results. Protocols.io assigns your protocol its own identifier (DOI) so that it can be cited independently in the future. For instructions see: https://journals.plos.org/plosone/s/submission-guidelines#loc-laboratory-protocols. Additionally, PLOS ONE offers an option for publishing peer-reviewed Lab Protocol articles, which describe protocols hosted on protocols.io. Read more information on sharing protocols at https://plos.org/protocols?utm_medium=editorial-email&utm_source=authorletters&utm_campaign=protocols.

We look forward to receiving your revised manuscript.

Kind regards,

Simon Porcher

Academic Editor

PLOS ONE

Journal Requirements:

When submitting your revision, we need you to address these additional requirements.

1. Please ensure that your manuscript meets PLOS ONE's style requirements, including those for file naming. The PLOS ONE style templates can be found at 

https://journals.plos.org/plosone/s/file?id=wjVg/PLOSOne_formatting_sample_main_body.pdf and 

https://journals.plos.org/plosone/s/file?id=ba62/PLOSOne_formatting_sample_title_authors_affiliations.pdf

2. We note that you have indicated that there are restrictions to data sharing for this study. PLOS only allows data to be available upon request if there are legal or ethical restrictions on sharing data publicly. For more information on unacceptable data access restrictions, please see http://journals.plos.org/plosone/s/data-availability#loc-unacceptable-data-access-restrictions. 

Before we proceed with your manuscript, please address the following prompts:

a) If there are ethical or legal restrictions on sharing a de-identified data set, please explain them in detail (e.g., data contain potentially identifying or sensitive patient information, data are owned by a third-party organization, etc.) and who has imposed them (e.g., a Research Ethics Committee or Institutional Review Board, etc.). Please also provide contact information for a data access committee, ethics committee, or other institutional body to which data requests may be sent.

b) If there are no restrictions, please upload the minimal anonymized data set necessary to replicate your study findings to a stable, public repository and provide us with the relevant URLs, DOIs, or accession numbers. For a list of recommended repositories, please see

https://journals.plos.org/plosone/s/recommended-repositories. You also have the option of uploading the data as Supporting Information files, but we would recommend depositing data directly to a data repository if possible.

We will update your Data Availability statement on your behalf to reflect the information you provide.

3. Please include captions for your Supporting Information files at the end of your manuscript, and update any in-text citations to match accordingly. Please see our Supporting Information guidelines for more information: http://journals.plos.org/plosone/s/supporting-information.

[Note: HTML markup is below. Please do not edit.]

Reviewers' comments:

Reviewer's Responses to Questions

**Comments to the Author**

1. Is the manuscript technically sound, and do the data support the conclusions?

The manuscript must describe a technically sound piece of scientific research with data that supports the conclusions. Experiments must have been conducted rigorously, with appropriate controls, replication, and sample sizes. The conclusions must be drawn appropriately based on the data presented. 

Reviewer #1: Yes

Reviewer #2: Partly

Reviewer #3: Yes

**********

2. Has the statistical analysis been performed appropriately and rigorously? 

Reviewer #1: Yes

Reviewer #2: Yes

Reviewer #3: Yes

**********

3. Have the authors made all data underlying the findings in their manuscript fully available?

The PLOS Data policy requires authors to make all data underlying the findings described in their manuscript fully available without restriction, with rare exception (please refer to the Data Availability Statement in the manuscript PDF file). The data should be provided as part of the manuscript or its supporting information, or deposited to a public repository. For example, in addition to summary statistics, the data points behind means, medians and variance measures should be available. If there are restrictions on publicly sharing data—e.g. participant privacy or use of data from a third party—those must be specified.

Reviewer #1: Yes

Reviewer #2: Yes

Reviewer #3: Yes

**********

4. Is the manuscript presented in an intelligible fashion and written in standard English?

PLOS ONE does not copyedit accepted manuscripts, so the language in submitted articles must be clear, correct, and unambiguous. Any typographical or grammatical errors should be corrected at revision, so please note any specific errors here.

Reviewer #1: Yes

Reviewer #2: Yes

Reviewer #3: Yes

**********

5. Review Comments to the Author

Please use the space provided to explain your answers to the questions above. You may also include additional comments for the author, including concerns about dual publication, research ethics, or publication ethics. (Please upload your review as an attachment if it exceeds 20,000 characters)

Reviewer #1: I enjoyed reading this paper as it addresses an important topic, uses rich data and sound methods, and presents interesting results. Hence, I am happy to be part of the review team.

I understand that the publication criteria at PLOS ONE do not require the authors to comprehensively review the literature, and the contribution of a paper to the literature is irrelevant. However, not citing an earlier study that does essentially the same but for a different country is unethical and would constitute plagiarism. Garz and Rickardsson (2023 “Ownership and media slant: Evidence from Swedish newspapers”, Kyklos; as a working paper available online since 2021) nearly has the same title, makes the same theoretical arguments, and uses the same method of text analysis. At a minimum, I recommend to reference the earlier paper, not least to avoid concerns about unethical behavior and plagiarism. Ideally, however, it would be nice to see a short discussion of how the submitted paper differs and how it adds to previous findings. For example, I think adding further evidence from another context (Australia) may help to generalize certain results; the focus on newspaper acquisitions is different; and using the synthetic difference-in-differences approach to study these mergers is novel and appealing.

More generally speaking, even though it might not be relevant for PLOS ONE, it seems that the paper would benefit from a proper literature review. While the reference list includes multiple up-to-date references to relevant non-scholarly work (e.g., Gallup polls or newspaper reports about acquisitions), with the exception of Bursztyn et al. (2023), the paper misses scholarly studies published during the past decade.

In conclusion, I thank the authors for taking on this interesting project and wish them success moving forward with the paper.

Reviewer #2: This is a solid paper. The topic is certainly important and the data analysis is impressive. The main results are plausible and interesting. There are a few issues which is why I answer "partly" re analysis being sound. Here are my main comment/suggestions:

-I don't know how well the paper addresses the question of whether bias is supply or demand driven, as claimed. Are the supply-side biases/leanings of the owners other than News Corps even discussed? Even their ideology is discussed very briefly, in just one sentence I think. I would discuss evidence or lack thereof of bias/ideology of each owner/ownership group, including owners who gave up ownership in each merger, in some earlier section before the results, perhaps the section describing the mergers. I also think a crucial variable is ignored in the analysis, the degree of competition each newspaper faces in its metropolitan area. Supply-side bias is much more likely to influence slant in a paper with a local monopoly. It would be ideal to see the paper account for this factor but I'm not sure if it's necessary for publication in this journal. (I don't insist on this for this journal.)

-The description of the 2019 merger seems inconsistent: the intro says "in 2019, Australian entrepreneur Antony Catalano bought Australian Community Media from Nine Entertainment" (who is he and what is his ideology?) and later the paper refers to "Australian Community Media’s Acquisition of Nine Entertainment’s 257

Regional Newspapers (2019)"

-I found Figures 4 and 5 very difficult to read. I'd get rid of gridlines, show or at least clearly say when the merger occurred, what each series is (there seem to be three shown but just two in the legend - the series at the bottom of each figure (shaded below in pinkish), which don't go all the way across, seem to not be labeled? And the slants for treatment and control groups are weirdly highly correlated, with many peaks and valleys occurring at the same time...

-The regression tables are also unclear. What is the unit of observation - a newspaper-quarter? How many observations are used in each model. These should be stated. If it is newspaper-quarter, I'm not sure if this is right since many newspapers with what should be similar slants over time will be repeated - wouldn't one observation for each newspaper before and after the merger period be better? If newspaper-quarter, should standard errors be clustered by newspaper to account for serial correlation? (Why jackknife std errors?) And tests of significant differences across models should be conducted with p-values shown, or the author could interact variables (like High Coalition) with the treatment to test if this leads to a significant diff.

-I found the introduction to need work, eg it is redundant - it shouldn't state results in the first paragraph and then again on the next page. I also found the reference to the "interquantile range" to be impossible to understand - which quantiles? This range is never referred to again later in the paper.

-The slant verification is quite nice and the lists of top trigrams indeed seem ideologically consistent. The method for measuring slant seems quite similar to Gentzkow and Shapiro (2010) but this is not stated clearly - the parts which are the same as the prior paper should be acknowledged, and innovations or differences should be described clearly too.

-Why 150 top trigrams? Perhaps robustness to other numbers should be shown.

Reviewer #3: This paper studies a policy relevant topic in a new and interesting setting. Understanding whether media slant is supply- or demand-driven is revenant for media regulators as market concentration can have opposite effects on slant depending on its source. The highly concentrated newspaper market in Australia, and the period of general decline of print media, present a revenant case study for answering this question.

The paper employs text analysis techniques to quantify slant, and a synthetic difference-in-difference approach to study its dynamics around ownership changes. Overall, the results suggest in the Australian case ownership changes have a significant effect on slant. This adds important nuance to the previous literature, which has focused mostly on the US, and has argued that the role of supply-side factors in determining media slant is relatively small.

Comments:

1. I have two comments on the validation of the text-based measure of slant (which is an important part of the paper).

a. First, the validation exercise focuses only the cross-sectional variation in slant across newspapers, rather than its time-varying component. Allowing the slant index to vary over time is the main innovation of this application relative to the original measure developed by Gentzkow-Shapiro 2010. Therefore, it think that the paper should spend more time discussing the time dimension, and should provide evidence (at least anecdotal) that it picks up meaningful over-time variation in the tone of reporting.

b. The slant index is shown to correlate with the share of Labor- vs Coalition-leaning readers. I think that this is reassuring, but different from the definition of “biased reporting” — a newspaper can be read by an audience with a given political orientation but be neutral in its reporting. The ideal validation would be against perceptions of --bias-- in reporting — e.g. a survey that asks people to position the newspaper on the spectrum of Labor-biased / neutral / Coalition-biased.

4. For transparency, I would recommend reporting the mix of newspapers picked up in the synthetic control method for each treated newspaper along with their respective weights.

It was also not entirely clear why the synthetic control was preferred over a more standard difference-in-difference method using all untreated newspapers as control units. If the concern are common trends in slant (as suggested by Figure 1), why not simply difference them out with time effects?

3. It would also be useful to report the baseline levels of the slant in each table and a give a better sense of the magnitude of the estimated effects and how they compare to the literature.

4. I think that the paper could provide more context on the political orientation of the owners of interest. This would be helpful for readers who are not familiar with the Australian market.

**********

6. PLOS authors have the option to publish the peer review history of their article (what does this mean?). If published, this will include your full peer review and any attached files.

If you choose “no”, your identity will remain anonymous but your review may still be made public.

**Do you want your identity to be public for this peer review?** For information about this choice, including consent withdrawal, please see our Privacy Policy.

Reviewer #1: No

Reviewer #2: No

Reviewer #3: No

**********

[NOTE: If reviewer comments were submitted as an attachment file, they will be attached to this email and accessible via the submission site. Please log into your account, locate the manuscript record, and check for the action link "View Attachments". If this link does not appear, there are no attachment files.]

While revising your submission, please upload your figure files to the Preflight Analysis and Conversion Engine (PACE) digital diagnostic tool, https://pacev2.apexcovantage.com/. PACE helps ensure that figures meet PLOS requirements. To use PACE, you must first register as a user. Registration is free. Then, login and navigate to the UPLOAD tab, where you will find detailed instructions on how to use the tool. If you encounter any issues or have any questions when using PACE, please email PLOS at figures@plos.org. Please note that Supporting Information files do not need this step.

# Author comment: Author response to Decision Letter 0

**DOI:** 10.1371/journal.pone.0315137.r002

9 Oct 2024

Our responses to reviews were attached as a file at the earlier step.

Attachment Submitted filename: Reponses to Referees.pdf

# Peer review: Decision Letter 1

**DOI:** 10.1371/journal.pone.0315137.r003

**Authors:** Simon Porcher

21 Nov 2024

Media Ownership and Ideological Slant: Evidence from Australian Newspaper Mergers

PONE-D-24-27523R1

Dear Dr. Volkova,

We’re pleased to inform you that your manuscript has been judged scientifically suitable for publication and will be formally accepted for publication once it meets all outstanding technical requirements.

Within one week, you’ll receive an e-mail detailing the required amendments. When these have been addressed, you’ll receive a formal acceptance letter and your manuscript will be scheduled for publication.

An invoice will be generated when your article is formally accepted. Please note, if your institution has a publishing partnership with PLOS and your article meets the relevant criteria, all or part of your publication costs will be covered. Please make sure your user information is up-to-date by logging into Editorial Manager at Editorial Manager® and clicking the ‘Update My Information' link at the top of the page. If you have any questions relating to publication charges, please contact our Author Billing department directly at authorbilling@plos.org.

If your institution or institutions have a press office, please notify them about your upcoming paper to help maximize its impact. If they’ll be preparing press materials, please inform our press team as soon as possible -- no later than 48 hours after receiving the formal acceptance. Your manuscript will remain under strict press embargo until 2 pm Eastern Time on the date of publication. For more information, please contact onepress@plos.org.

Kind regards,

Simon Porcher

Academic Editor

PLOS ONE

Additional Editor Comments (optional):

Reviewers' comments:

Reviewer's Responses to Questions

**Comments to the Author**

1. If the authors have adequately addressed your comments raised in a previous round of review and you feel that this manuscript is now acceptable for publication, you may indicate that here to bypass the “Comments to the Author” section, enter your conflict of interest statement in the “Confidential to Editor” section, and submit your "Accept" recommendation.

Reviewer #1: All comments have been addressed

Reviewer #2: All comments have been addressed

Reviewer #3: (No Response)

**********

2. Is the manuscript technically sound, and do the data support the conclusions?

The manuscript must describe a technically sound piece of scientific research with data that supports the conclusions. Experiments must have been conducted rigorously, with appropriate controls, replication, and sample sizes. The conclusions must be drawn appropriately based on the data presented. 

Reviewer #1: Yes

Reviewer #2: Yes

Reviewer #3: (No Response)

**********

3. Has the statistical analysis been performed appropriately and rigorously? 

Reviewer #1: Yes

Reviewer #2: Yes

Reviewer #3: (No Response)

**********

4. Have the authors made all data underlying the findings in their manuscript fully available?

The PLOS Data policy requires authors to make all data underlying the findings described in their manuscript fully available without restriction, with rare exception (please refer to the Data Availability Statement in the manuscript PDF file). The data should be provided as part of the manuscript or its supporting information, or deposited to a public repository. For example, in addition to summary statistics, the data points behind means, medians and variance measures should be available. If there are restrictions on publicly sharing data—e.g. participant privacy or use of data from a third party—those must be specified.

Reviewer #1: Yes

Reviewer #2: Yes

Reviewer #3: (No Response)

**********

5. Is the manuscript presented in an intelligible fashion and written in standard English?

PLOS ONE does not copyedit accepted manuscripts, so the language in submitted articles must be clear, correct, and unambiguous. Any typographical or grammatical errors should be corrected at revision, so please note any specific errors here.

Reviewer #1: Yes

Reviewer #2: Yes

Reviewer #3: (No Response)

**********

6. Review Comments to the Author

Please use the space provided to explain your answers to the questions above. You may also include additional comments for the author, including concerns about dual publication, research ethics, or publication ethics. (Please upload your review as an attachment if it exceeds 20,000 characters)

Reviewer #1: (No Response)

Reviewer #2: The authors seem to have done a good job of responding to all comments. Congratulations on the completion of a thoughtful and interesting research project.

Reviewer #3: (No Response)

**********

7. PLOS authors have the option to publish the peer review history of their article (what does this mean?). If published, this will include your full peer review and any attached files.

If you choose “no”, your identity will remain anonymous but your review may still be made public.

**Do you want your identity to be public for this peer review?** For information about this choice, including consent withdrawal, please see our Privacy Policy.

Reviewer #1: No

Reviewer #2: No

Reviewer #3: No

**********

# Editor report: Acceptance letter

**DOI:** 10.1371/journal.pone.0315137.r004

**Authors:** Simon Porcher

16 Dec 2024

PONE-D-24-27523R1 

PLOS ONE

Dear Dr. Volkova, 

I'm pleased to inform you that your manuscript has been deemed suitable for publication in PLOS ONE. Congratulations! Your manuscript is now being handed over to our production team.

At this stage, our production department will prepare your paper for publication. This includes ensuring the following:

* All references, tables, and figures are properly cited

* All relevant supporting information is included in the manuscript submission,

* There are no issues that prevent the paper from being properly typeset

If revisions are needed, the production department will contact you directly to resolve them. If no revisions are needed, you will receive an email when the publication date has been set. At this time, we do not offer pre-publication proofs to authors during production of the accepted work. Please keep in mind that we are working through a large volume of accepted articles, so please give us a few weeks to review your paper and let you know the next and final steps. 

Lastly, if your institution or institutions have a press office, please let them know about your upcoming paper now to help maximize its impact. If they'll be preparing press materials, please inform our press team within the next 48 hours. Your manuscript will remain under strict press embargo until 2 pm Eastern Time on the date of publication. For more information, please contact onepress@plos.org.

If we can help with anything else, please email us at customercare@plos.org.

Thank you for submitting your work to PLOS ONE and supporting open access. 

Kind regards, 

PLOS ONE Editorial Office Staff

on behalf of

Pr. Simon Porcher 

Academic Editor

PLOS ONE
